# Exploring atypical spatial-functional coupling in adolescent autism spectrum disorder: insights from neurodevelopment and transcriptomic architecture

**DOI:** 10.3389/fnins.2026.1780430

**Published:** 2026-07-27

**Authors:** Jun Pan, Heng Zhang, Yiran Zhai, Jiang Zhang, Hanbin Deng

**Affiliations:** 1 College of Electrical Engineering, Sichuan University, Chengdu, China; 2 Sichuan Institute of Computer Sciences, Chengdu, China

**Keywords:** autism spectrum disorder, distance-dependent functional connectivity, multiscale brain organization, resting-state fMRI, variogram analysis

## Abstract

Autism Spectrum Disorder (ASD) is associated with atypical large-scale brain network organization, yet how spatial-functional dependencies relate to clinical features and molecular reference maps remains incompletely understood. To quantify spatial functional heterogeneity (Sill) and coherence persistence (Range), we analyzed resting-state fMRI data from 162 ASD and 175 TD adolescents, all aged 12–18. Compared with TD, adolescents with ASD exhibited significantly increased Sill within higher-order association networks, including the left Language and right Posterior Multimodal networks, whereas no group differences in Range survived multiple-comparison correction. Within the ASD group, elevated Sill was selectively associated with greater social-affective symptom severity but not restricted and repetitive behaviors. To explore potential biological correlates, we integrated cortical gene expression reference data and identified transcriptomic patterns associated with regional Sill differences. These genes showed enrichment for synaptic signaling, mitochondrial processes, and glial-related functions, highlighting multiscale correspondence between spatial-functional organization and molecular reference maps. Together, these results demonstrate statistical associations among altered spatial-functional properties, clinical severity, and transcriptomic profiles related to synaptic signaling, mitochondrial processes, and glial-related functions in ASD, providing a complementary spatial perspective on large-scale functional organization.

## Introduction

Autism spectrum disorder (ASD) is a neurodevelopmental condition characterized by persistent differences in social communication and restricted or repetitive behaviors, accompanied by substantial heterogeneity in behavioral presentation and neurobiological organization (Lord et al., 2020). Neuroimaging studies have increasingly emphasized that ASD involves distributed alterations across large-scale brain systems rather than focal abnormalities confined to isolated regions. Resting-state functional magnetic resonance imaging (MRI) has played a central role in revealing atypical functional organization in ASD, particularly within association cortices supporting higher cognitive and social processes (Hull et al., 2016).

Recent large-scale neuroimaging initiatives further suggest that inter-individual variability in brain network organization represents an important dimension of brain organization in neurodevelopmental conditions. Rather than reflecting uniform deficits, ASD-related neural differences appear to involve altered patterns of inter-individual variability and regional specialization across association cortex, highlighting the importance of approaches capable of characterizing spatial organization beyond average connectivity measures (Nomi and Uddin, 2015).

Early work highlighted disrupted functional connectivity as a prominent feature of ASD, including altered long-range synchronization and network integration (Just et al., 2004). Subsequent studies demonstrated that atypical connectivity patterns extend across multiple canonical functional systems, including language, default-mode, and social cognitive networks (Di Martino et al., 2014). Large multisite datasets further confirmed that functional alterations in ASD are spatially heterogeneous and vary across cortical territories (Nunes et al., 2019). These findings suggest that characterizing spatial organization of functional signals may provide complementary information beyond conventional connectivity analyses. Indeed, emerging work has emphasized that functional brain organization can also be described in terms of spatial embedding, whereby functional similarity follows structured spatial constraints across the cortical sheet. Such spatial perspectives complement network-based models by capturing how functional signals vary continuously across cortex rather than being restricted to discrete regions or connections (Alexander-Bloch et al., 2013).

In systems neuroscience, variogram-based approaches have emerged as a powerful tool for evaluating complex systems, offering quantitative metrics for spatial variability and continuity. By applying these methods to neuroimaging data, researchers can assess spatial heterogeneity without being restricted by predefined network boundaries (Burt et al., 2020). Central to this framework are two key parameters: the Sill, which captures the total variance magnitude (with higher values reflecting pronounced spatial heterogeneity or dissimilarity among distant regions), and the Range, which defines the correlation distance (where larger values indicate smoother, more gradual transitions of cortical properties). Because these spatial statistical frameworks provide atlas-free descriptors of spatial autocorrelation, they are increasingly valued for revealing the intrinsic spatial structure of brain activity, thereby serving as a vital complement to traditional connectomic analyses (Markello and Misic, 2021). This alternative perspective is particularly relevant given recent findings that intrinsic brain activity exhibits structured spatial dependencies across the cortical surface, demonstrating how functional similarity evolves with spatial distance.

Emerging evidence indicates that association cortices show greater inter-individual variability in brain network organization compared to primary sensory regions (Mueller et al., 2013). These regions are also preferentially implicated in ASD across structural and functional imaging studies (Ecker et al., 2015). However, it remains unclear whether ASD involves altered spatial dependence of functional activity itself, rather than solely altered connectivity strength.

In addition to large-scale alterations in brain organization, previous neuroimaging studies have reported associations between functional connectivity patterns and clinical symptom severity in ASD. In particular, altered connectivity within networks supporting social cognition, including the default mode network and related social brain systems, has been linked to social-affective symptom severity in individuals with ASD (Kennedy and Courchesne, 2008). Consistent with this view, atypical functional interactions involving medial prefrontal and temporoparietal regions have been associated with impairments in social communication and social cognitive processing (Just et al., 2012). Resting-state studies have further demonstrated that disruptions in large-scale functional networks are related to variability in social-affective symptoms across individuals with ASD (Hull et al., 2017). By contrast, associations between functional connectivity patterns and restricted and repetitive behaviors (RRB) have been less consistently observed across studies (Uddin et al., 2013). Large-scale collaborative datasets have also highlighted substantial heterogeneity in brain network organization in ASD, suggesting that neural variability may contribute to differences in clinical symptom profiles (Di Martino et al., 2014). Together, these findings suggest that variability in large-scale brain organization may differentially relate to distinct clinical dimensions of ASD, highlighting the importance of examining relationships between brain organization and clinical symptom severity.

Linking macroscale imaging patterns to biological reference maps has become an important strategy for interpreting neuroimaging findings. Imaging transcriptomics studies have demonstrated spatial correspondence between functional brain organization and regional gene expression profiles (Hawrylycz et al., 2012). Integrative analyses further suggest that neurotransmitter receptor distributions provide an additional molecular framework constraining large-scale functional architecture (Hansen et al., 2022). Such cross-scale approaches enable statistical examination of whether macroscale imaging alterations align with molecular and cellular organization. Importantly, cross-scale integration frameworks do not assume direct mechanistic correspondence but instead test whether independently derived biological reference maps exhibit convergent spatial organization with imaging-derived patterns. This strategy has increasingly been adopted to contextualize macroscale neuroimaging findings within broader neurobiological systems (Fornito et al., 2019).

Despite growing progress, three major questions remain unresolved. First, whether ASD is associated with altered spatial-functional dependency properties across the cortex has not been systematically examined. Second, it remains unclear whether spatial alterations preferentially localize to specific large-scale functional systems. Third, the extent to which these spatial patterns correspond to clinical symptom severity and molecular reference maps remains insufficiently characterized.

**FIGURE 1:**
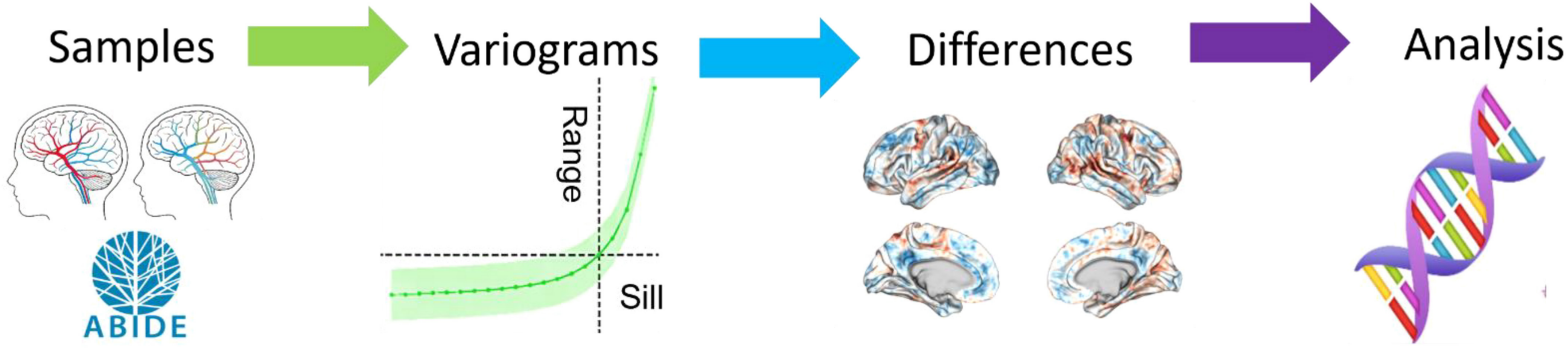
Schematic overview of the analytical pipeline. This workflow integrates neuroimaging and molecular data to elucidate ASD-related functional alterations through four sequential stages: (1) Samples, where whole-brain functional connectivity matrices were constructed using fMRI data from the ABIDE consortium (ASD and TD controls); (2) Variogram Modeling, which utilized BrainSMASH to compute empirical variograms based on geodesic distances, yielding the Range (the spatial distance at which functional similarity decays to baseline, representing the scale of local functional organization) and the Sill (the asymptotic variance reflecting maximum functional heterogeneity across the cortex); (3) Group Differences, where spatial patterns of functional connectivity alterations were mapped onto the cortical surface and validated using spin tests to account for spatial autocorrelation; and (4) Molecular Basis, in which PLS regression was employed to link these spatial difference maps with neurotransmitter receptor distributions and gene expression profiles to reveal the potential molecular underpinnings of ASD.

To address these questions, we quantified spatial-functional dependency using variogram-derived metrics in a large resting-state fMRI cohort of adolescents with ASD and TD controls. We examined group differences, associations with symptom severity, network-level distributions, and correspondence with neurotransmitter and transcriptomic reference datasets. This multilevel framework provides a spatially informed characterization of functional organization in ASD while avoiding assumptions about predefined connectivity architecture. Importantly, this framework is designed to characterize spatial-functional organization at the group level and does not aim to establish clinically actionable biomarkers. The overall design of the study is summarized in Figure 1.

## Results

### ASD-TD group differences in spatial-functional dependency metrics

**FIGURE 2:**
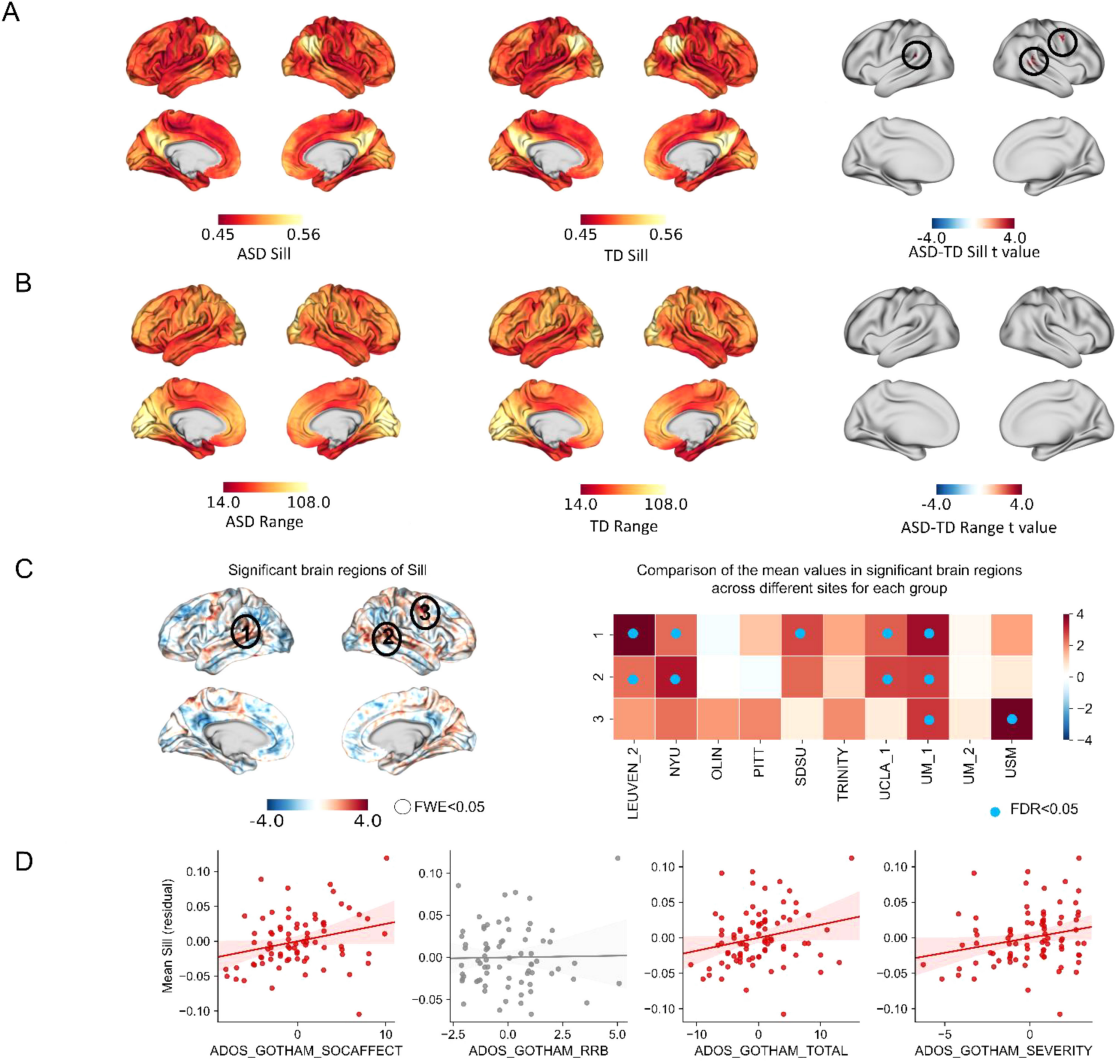
ASD–TD differences in spatial-functional dependency metrics and associations with symptom severity. **(A)** Group-averaged cortical maps of Sill for ASD and TD participants, and the corresponding ASD–TD Sill T-statistic map. The ASD–TD comparison was performed using vertex-wise two-sample *t*-tests; significant clusters are shown after family-wise error (FWE) correction (*p* <0.05). **(B)** Group-averaged cortical maps of Range for ASD and TD participants, and the corresponding ASD–TD Range T-statistic map. No clusters survived multiple-comparison correction for Range. **(C)** Left: Cortical regions showing significant ASD–TD differences in Sill after FWE correction, primarily localized to bilateral temporal cortices and right motor cortex (labeled 1, 2, 3). Right: Mean Sill values were extracted from the combined significant clusters as a single unified ROI and are shown across acquisition sites. Scanning site was included as a covariate in all statistical models to control for site-related variability. The direction of group differences (ASD > TD) was consistent across sites; blue dots indicate effects surviving FDR correction (*P*_FDR_ < 0.05). Effect sizes (Cohen’s d) for the significant clusters are reported in Supplementary Table 5. **(D)** Associations between mean Sill values extracted from the combined significant clusters (as a single unified ROI) and ADOS symptom measures within the ASD group. Partial correlations were computed controlling for age, sex, site, and mean FD; error bars indicate 95% confidence intervals. The association with Social Affect survived FDR correction [*r* = 0.30, 95% CI (0.08, 0.49), *P_*FDR*_* = 0.037]. Associations with Total [*r* = 0.25, 95% CI (0.03, 0.44), *p* = 0.027, *P_*FDR*_* = 0.051] and Calibrated Severity [*r* = 0.23, 95% CI (0.01, 0.43), *p* = 0.039, *P_*FDR*_* = 0.051] reached nominal significance only and did not survive FDR correction. No significant association was found with Restricted and Repetitive Behavior scores [*r* = –0.06, 95% CI (–0.29, 0.17), *p* = 0.611].

We first computed vertex-wise Sill and Range values across the cortical surface for each participant and examined ASD-TD differences, whereas no clusters survived multiple-comparison correction for Range. We identified three brain regions with significantly enhanced Sill in adolescents with autism, primarily involving bilateral temporal regions (left: *P*_*FWE*_ < 0.001, Cohen’s *d* = 0.53; right: *P*_*FWE*_ < 0.001, Cohen’s *d* = 0.50) and the right motor cortex (*P*_*FWE*_ < 0.001, Cohen’s *d* = 0.58) (Figure 2 and Supplementary Table 1).

To evaluate robustness, mean Sill values were extracted from the combined significant clusters as a single unified ROI for visualization across acquisition sites. Scanning site was included as a covariate in all statistical models to account for site-related variability. Across all cohorts, ASD subjects exhibited consistently higher mean Sill than TD subjects (Figure 2, right heatmap). This consistency confirmed that observed alterations were not attributable to site-specific variability.

We further assessed whether head motion, indexed by mean framewise displacement (FD), was associated with our key metrics within the regions showing significant ASD–TD differences. No significant correlations were observed between FD and Range [*r* = 0.09, *p* = 0.25, 95% CI = (–0.065, 0.241)], FD and Sill [*r* = 0.04, *p* = 0.59, 95%, CI = (–0.115, 0.193)], FD and cortical thickness [*r* = 0.07, *p* = 0.339, 95% CI = (–0.085, 0.222)], or FD and any Autism Diagnostic Observation Schedule (ADOS)-Gotham clinical symptom scores. Whole-brain vertex-wise correlation analyses presented in Supplementary Figure 1 further demonstrate that while scattered associations between FD and Range, Sill, and cortical thickness were observed before correction, no clusters surviving FDR correction overlapped with our main ASD–TD difference maps, confirming that head motion did not confound or drive the observed group differences.

Furthermore, to account for potential site-specific variance and ensure the robustness of our findings, we conducted a sensitivity analysis using a linear mixed-effects model with site modeled as a random effect. This analysis confirmed that the observed Sill alterations in ASD remained statistically significant (cluster-level *P* <0.001; Supplementary Figure 2), indicating that our results are robust to the heterogeneity introduced by multisite data acquisition.

### Comparison with conventional rs-fMRI metrics

To evaluate whether variogram-derived metrics capture information beyond established rs-fMRI measures, we compared the spatial patterns of group differences (ASD vs. TD) derived from Sill and Range with those obtained from several widely used indices, including ReHo, ALFF, and degree centrality (DC). Specifically, we computed vertex-wise t-statistic maps for each conventional metric and assessed their spatial correlations with the Sill and Range t-maps. The results showed that the spatial distributions of Sill- and Range-related alterations exhibited weak correlations with the patterns observed for these conventional measures (Supplementary Figure 3). These findings suggest that variogram-derived metrics are not reducible to local synchronization, spontaneous activity amplitude, or network centrality measures, and therefore capture a partially distinct aspect of cortical functional organization.

### Association between altered regions of Sill and symptom severity

Within the ASD group, we assessed associations between Sill values in the significant clusters and clinical symptom severity using partial correlation analyses controlling for age, sex, site, and mean FD.

After covariate adjustment, Sill values showed a significant positive association with ADOS Social Affect scores [*r* = 0.30, *P* = 0.009, *P*_*FDR*_ = 0.037, 95% CI (0.08, 0.488)]. Associations with ADOS Total scores [*r* = 0.25, *P* = 0.027, *P*_*FDR*_ = 0.051, 95% CI (0.03, 0.44)] and ADOS Calibrated Severity scores [*r* = 0.23, *P* = 0.039, *P*_*FDR*_ = 0.051, 95% CI (0.01, 0.43)] did not survive FDR correction and are therefore interpreted as exploratory trends. No significant association was observed between Sill values and Restricted and Repetitive Behavior (RRB) scores [*r* = –0.06, *P* = 0.611, 95% CI (–0.29, 0.17)] (Figure 2D and Supplementary Table 2). These findings suggest statistically dissociable associations of Sill with social-affective versus repetitive symptoms in adolescents with ASD. However, with modest effect sizes (*r* = 0.30) explaining < 10% of the variance, these associations should be interpreted as preliminary evidence of potential brain-behavior relationships rather than as definitive clinical substrates. Independent replication in larger cohorts is necessary before drawing translational conclusions.

### 12 Network-level distribution and functional characterization of Sill alterations

**FIGURE 3:**
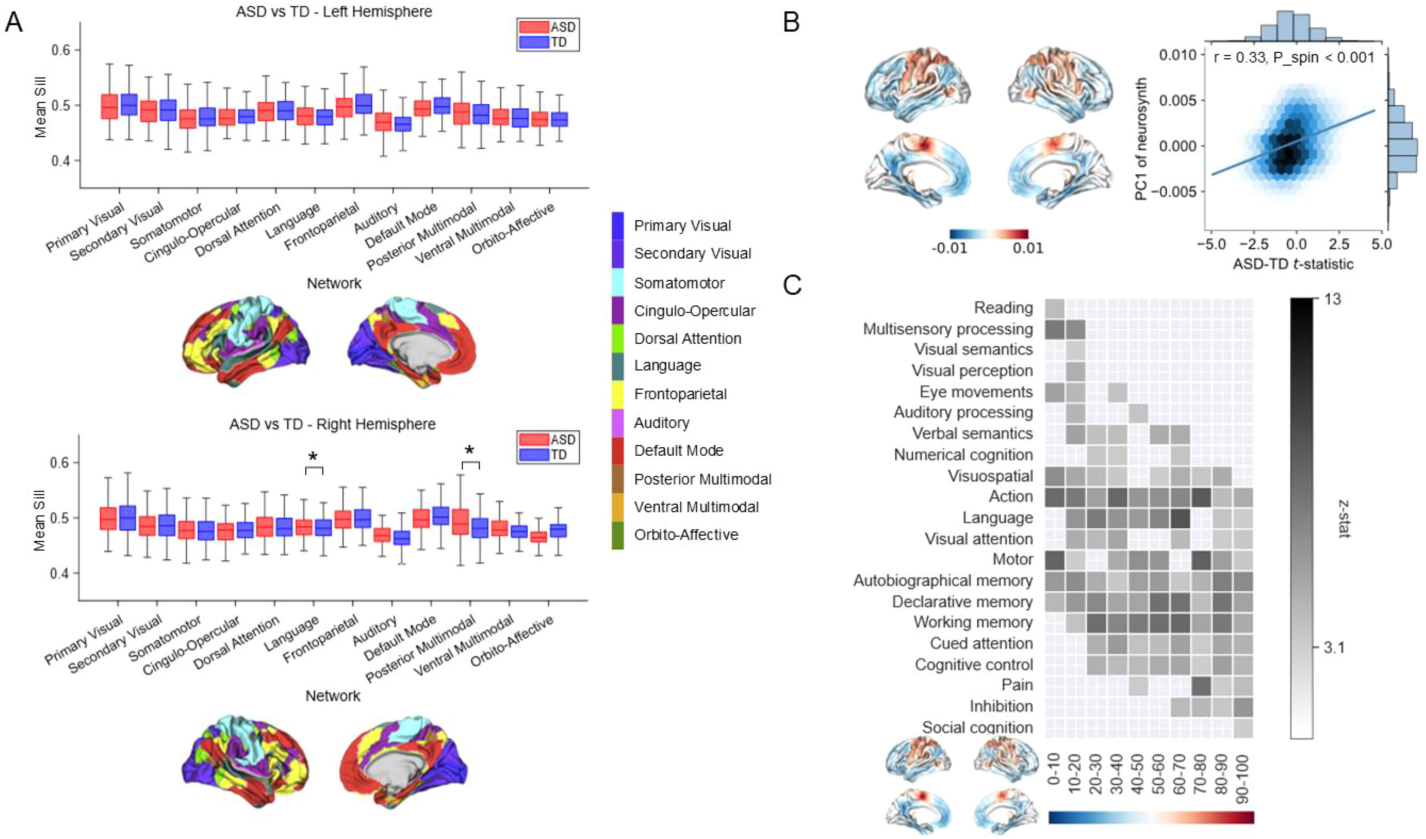
Network-level distribution and cognitive decoding of ASD-related Sill alterations. **(A)** Network-level Sill differences between ASD and TD groups. Vertex-wise Sill values were averaged within each of the 12 canonical functional networks separately for the left and right hemispheres. Boxplots represent the distribution of mean Sill values across participants within each group. Asterisks indicate significant between-group differences. FDR correction was applied across networks within each hemisphere. Asterisks indicate FDR-corrected *p* <0.05. **(B)** Spatial association between ASD–TD Sill difference map and the principal cognitive component derived from Neurosynth meta-analytic terms. The vertex-wise ASD–TD t-statistic map was correlated with the first principal component (PC1) extracted from 123 cognitive terms. Significance was assessed using spin permutation testing to account for spatial autocorrelation (*r* = 0.33, *P*__spin_ < 0.001). **(C)** Functional decoding of ASD–TD Sill difference map. The group-difference T-statistic map was submitted to meta-analytic functional decoding using Neurosynth-derived cognitive term maps. The heatmap displays the strength of association (Z-statistics) between the ASD-related Sill alteration pattern and cognitive domains. Darker shades indicate stronger spatial correspondence.

To determine whether ASD-related Sill alterations were preferentially localized to specific large-scale systems, we summarized Sill values within each of the 12 canonical functional networks. Network-level comparisons revealed significant group differences in the Language and Posterior Multimodal networks after correction for multiple comparisons (Figure 3A). All 12 canonical networks were explicitly included in the network-level comparisons. In addition to primary sensory networks, other networks including the cingulo-opercular, dorsal attention, ventral multimodal, and orbit-affective networks also did not survive correction for multiple comparisons. Only the Language and Posterior Multimodal networks showed significant group differences after correction. These findings indicate that ASD-related Sill alterations are concentrated within a subset of higher-order functional systems rather than being uniformly distributed across the cortex.

### Cognitive and functional associations of cortical alterations

To further characterize the spatial organization of these effects, we assessed the association between the ASD–TD Sill difference map and the first principal component (PC1) derived from 123 meta-analytic cognitive terms (e.g., language, social cognition, working memory, semantics). A significant positive spatial correlation was observed (*r* = 0.33, *P*_*spin*_ < 0.001; Figure 3B).

Subsequently, we performed a functional decoding analysis based on 24 representative thematic terms provided in the study by Preti et al. (2017). Functional decoding of the ASD-TD Sill difference map was performed using 24 cognitive topic terms derived from the Neurosynth platform, covering domains including language, multisensory processing, visual semantics, working memory, and social cognition (Figure 3C). These terms were predominantly associated with associative and multimodal cortical territories, consistent with the network-level findings.

### Neurotransmitter model of ASD-related Sill alterations

**FIGURE 4:**
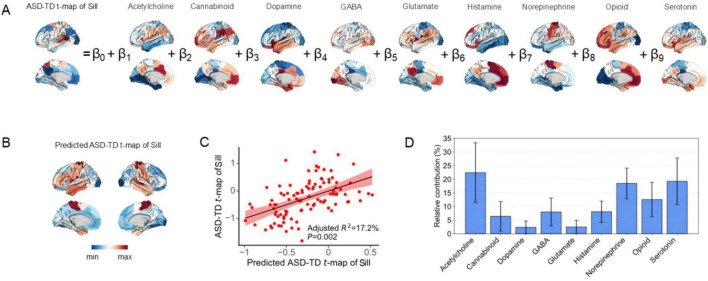
Neurotransmitter-associated modeling of ASD–TD Sill difference patterns. **(A)** A multiple linear regression model was constructed to examine whether spatial distributions of nine neurotransmitter systems (acetylcholine, cannabinoid, dopamine, GABA, glutamate, histamine, norepinephrine, opioid, and serotonin) jointly explain variance in the vertex-wise ASD–TD Sill difference map. Each neurotransmitter map was entered as an independent predictor. **(B)** The predicted ASD–TD Sill difference map derived from the regression model is shown, illustrating the spatial pattern reconstructed by the combined neurotransmitter predictors. **(C)** Model performance was evaluated by correlating predicted and empirical ASD–TD Sill difference values across cortical vertices. The scatterplot shows the spatial correspondence between predicted and observed maps (adjusted *R*^2^ and *p*-value indicated). **(D)** Neurotransmitter systems jointly shape the regression model, with each system’s contribution quantified as its normalized absolute standardized coefficient (divided by the sum across all nine systems). Error bars indicate 95% bootstrap confidence intervals (10,000 resamples). Asterisk indicates the predictor surviving FDR correction (*P*_FDR_ < 0.05). Corresponding statistics are provided in Supplementary Table 2. Residual plots and predictor correlation matrix are provided in Supplementary Figures 4, 7, respectively.

To examine whether the spatial distribution of ASD–TD Sill differences is statistically associated with cortical neurotransmitter architecture, we constructed a multiple linear regression model in which the ASD–TD Sill T-statistic map was predicted by nine neurotransmitter receptor/transporter density maps (Figure 4A).

The regression model generated a predicted Sill difference map that recapitulated the large-scale spatial pattern observed in the empirical ASD-TD T-statistic map (Figure 4B). To quantify model performance, we assessed the spatial correlation between the predicted and empirical maps. A significant positive association was observed (*r* = 0.30, *P* = 0.002; Figure 4C), with the model accounting for 17.2% of the variance in the ASD–TD Sill difference map (adjusted *R*^2^ = 0.172). This modest explanatory power is comparable to that reported in previous large-scale receptor-based spatial association studies (Li et al., 2024; Zhang et al., 2026), suggesting that additional biological, developmental, and environmental factors likely contribute to the observed spatial heterogeneity.

We next quantified the relative contribution of each neurotransmitter system to the regression model (Figure 4D and Supplementary Table 3). Norepinephrine emerged as the only neurotransmitter system that survived FDR correction [Relative contribution of 18.6%, *P*_*FDR*_ = 0.009, 95%CI (0.18, 0.75)]. Although acetylcholine showed the largest relative contribution [22.6%, 95% CI (–0.12, –0.02), *p* = 0.043], followed by serotonin [19.4%, 95% CI (0.06, 0.92), *p* = 0.027] and histamine [8.2%, 95% CI (–0.40, –0.01), *p* = 0.040], these associations did not survive FDR correction (*P*_*FDR*_ > 0.05). The remaining neurotransmitter systems (cannabinoid, dopamine, GABA, glutamate, opioid) showed non-significant contributions (*P*_*FDR*_ > 0.05, Figure 4D). Together, these results indicate that, among the nine neurotransmitter systems examined, norepinephrine is the most robust predictor of the spatial organization of ASD-associated Sill differences, with acetylcholine, serotonin, and histamine showing nominally significant but less stable associations. Residual plots for the regression model are provided in Supplementary Figure 4.

### Transcriptional correlates of ASD-related Sill alterations

**FIGURE 5:**
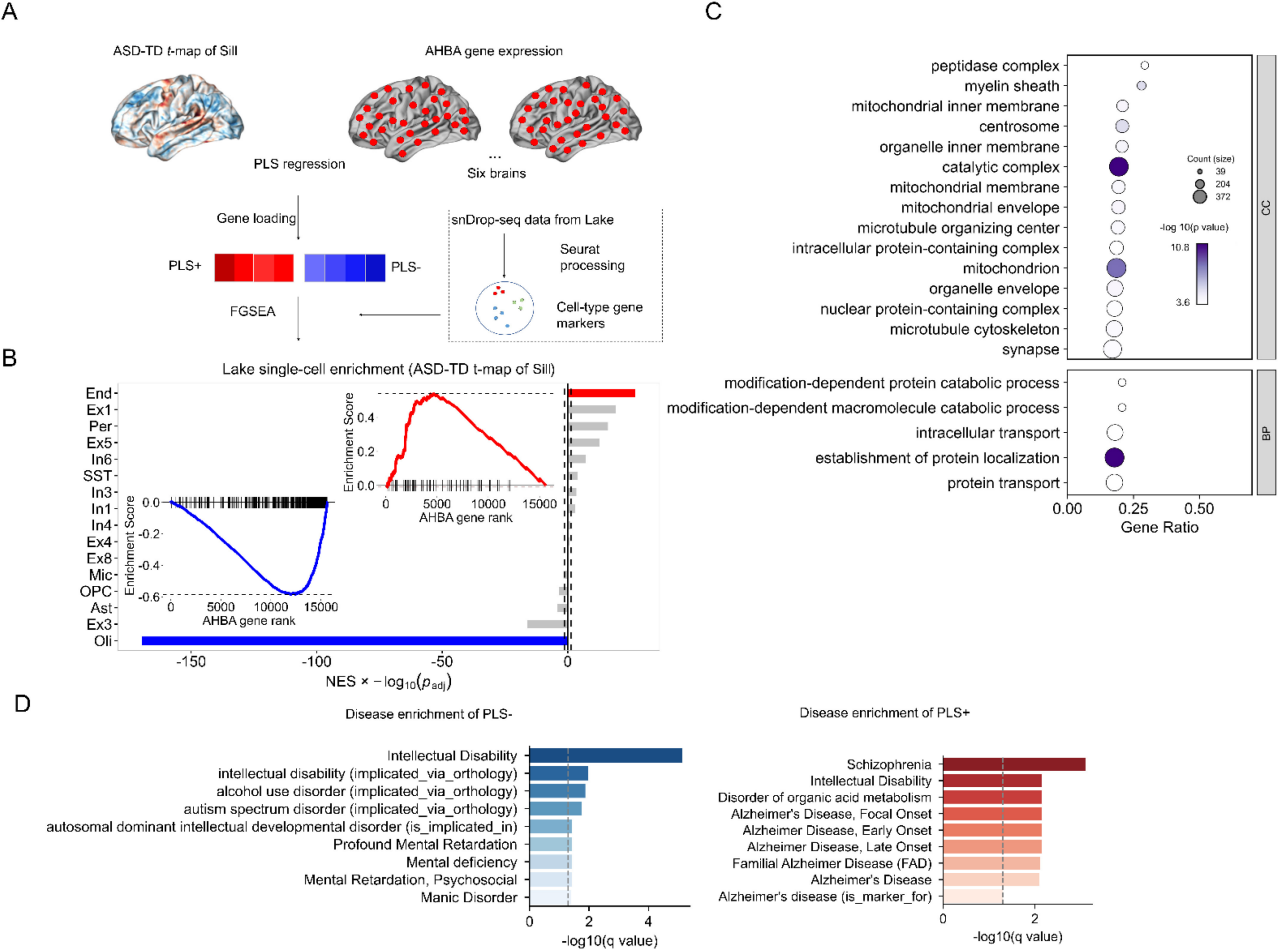
Transcriptomic associations of ASD–TD Sill difference patterns. **(A)** Partial least squares (PLS) regression was performed to examine the spatial association between the ASD–TD Sill difference map and cortical gene expression profiles derived from the Allen Human Brain Atlas (AHBA). Gene weights from significant PLS components (PLS+ and PLS–) were extracted for downstream enrichment analyses. **(B)** Cell-type enrichment analysis of PLS-weighted genes using single-nucleus RNA sequencing data from the Lake dataset. Gene set enrichment analysis (GSEA) revealed that PLS+ genes were significantly enriched in the End (endothelial) group, while PLS- genes were significantly enriched in the Oli (oligodendrocyte) group. Color indicates the direction of the enrichment score (positive: red; negative: blue), reflecting positive or negative spatial correspondence between cell type–specific gene expression patterns and ASD-related Sill alterations. **(C)** Gene ontology (GO) enrichment analysis of PLS-associated genes. The bubble plots display significantly enriched biological processes (BP) and cellular components (CC). Dot size represents gene count, with the three grey dots in the bottom right serving as a reference scale. Color indicates the direction of contribution weights. **(D)** Disease enrichment analysis of PLS-associated genes. Enriched disease terms are shown separately for positively and negatively weighted gene sets. Notably, several neurodevelopmental and intellectual disability–related categories were identified.

To investigate the transcriptional architecture associated with the spatial pattern of ASD-TD Sill differences, we performed PLS regression linking the ASD–TD Sill T-statistic map to cortical gene expression profiles from the Allen Human Brain Atlas (AHBA; Figure 5A). PLS1 explained 35% of the covariance between gene expression and ASD-TD Sill differences, a proportion consistent with prior imaging transcriptomic studies. The scree plot of variance explained across all PLS components (Supplementary Figure 5) confirms that PLS1 represents the dominant latent component, with subsequent components explaining substantially less variance.

We next characterized the cellular specificity of these gene sets using single-nucleus RNA sequencing data from the Lake dataset. Gene set enrichment analysis revealed that PLS+ genes showed significant enrichment in endothelial cells (End), while PLS- genes were significantly enriched in oligodendrocytes (Oli) (Figure 5B). The positive or negative enrichment described here implies a positive or negative spatial correspondence between the expression patterns of AHBA-derived cell type-specific genes and the ASD-related Sill alterations.

To further examine the biological functions represented in the PLS-associated genes, we conducted Gene Ontology (GO) enrichment analysis (Figure 5C). For PLS+ genes, the most significantly enriched cellular component (CC) terms included mitochondrial inner membrane (*P*_*FDR*_ = 1.2 × 10^–6^, gene ratio = 0.32), mitochondrion (*P*_*FDR*_ = 3.5 × 10^–5^, gene ratio = 0.28), and intracellular protein-containing complex (*P*_*FDR*_ = 8.7 × 10^–5^, gene ratio = 0.25). The top enriched biological process (BP) term was protein transport (*P*_*FDR*_ = 2.1 × 10^–4^, gene ratio = 0.29). Overall, the findings centered on a few key biological processes, including organelle biogenesis and protein transport, mitochondrial function, cytoskeletal organization, and synaptic structures.

Finally, disease enrichment analysis demonstrated that PLS+ genes were significantly overrepresented among gene sets implicated in neurodevelopmental and neuropsychiatric conditions, with the strongest enrichment observed for intellectual disability [-log_10_(q) = 4.82, *P*_*FDR*_ < 0.001] and schizophrenia [-log_10_(q) = 3.76, *P*_*FDR*_ = 0.002] (Figure 5D, left). In contrast, PLS- genes showed significant enrichment for Alzheimer’s disease-related gene sets (Figure 5D, right), consistent with their oligodendrocyte-related expression specificity. All disease enrichment results were FDR-corrected and validated with spin-based permutation testing (*P*_*spin*_ < 0.05).

### Robustness analysis

To verify the robustness of our findings under different methodological choices, we conducted a series of replication and extension analyses.

To evaluate whether unaccounted variance in handedness or IQ influenced the observed group differences in Sill, we performed supplementary sensitivity analyses restricted to participants with complete data on both covariates. Even after excluding participants with missing values at some sites, the core pattern of significantly elevated Sill in the ASD group remained largely unchanged and spatially consistent (Supplementary Figure 6). Specifically, when controlling for handedness, significant associations with Sill were localized to small, scattered regions in the temporal and parietal cortex. Similarly, when controlling for IQ, the pattern of associations was most prominent in the left temporal cortex and adjacent parietal regions, mirroring the spatial distribution of effects observed in the primary analysis. These results demonstrate that the main findings are robust to potential confounding effects of handedness and IQ, and the reduced sample size due to missing covariate data did not fundamentally alter the interpretation of the results.

To assess the robustness of these findings against potential multicollinearity among neurotransmitter receptor maps, we computed pairwise correlation matrices and variance inflation factors (VIFs) for the nine predictors. These analyses revealed moderate-to-high interdependence among several neurotransmitter systems (VIF range: 2.68–8.09), consistent with their known biological co-organization across the cortex (Supplementary Table 4 and Supplementary Figure 7).

To investigate the contribution of neurotransmitter systems to the observed spatial pattern of ASD-related alterations while minimizing the influence of multicollinearity among neurotransmitter maps, we performed ridge regression analyses (Supplementary Table 5 and Supplementary Figure 8).

To address the potential arbitrariness of selecting the upper and lower 10% gene expression thresholds, we further repeated the enrichment analyses using alternative cutoffs (top 5% and top 15%). Across these different thresholds, the enriched gene ontology terms showed high consistency with those identified using the original 10% gene set. In particular, the results converged on several major biological themes, including organelle assembly and protein transport, mitochondrial function, cytoskeletal organization, and synapse-related structures (Supplementary Figure 9). Notably, the myelin sheath category remained significantly enriched across all three thresholding schemes, further supporting the robustness and biological stability of our findings.

## Discussion

The present study investigated spatial-functional dependency properties of cortical activity in ASD using variogram-based metrics and multimodal reference mapping. Across analyses, ASD-related differences were primarily reflected in Sill rather than Range, reflecting altered spatial-functional heterogeneity rather than generalized changes in spatial extent. Significant effects were localized to bilateral temporal regions and the right motor cortex, suggesting regionally specific alterations rather than global cortical disruption. These findings extend prior connectivity-based studies by demonstrating that ASD-related alterations are observable not only in interregional coupling but also in the spatial structure of intrinsic functional signals themselves. This distinction suggests that alterations in ASD may involve statistical properties of how functional activity varies across cortical space, providing a complementary dimension to established connectivity abnormalities reported in the literature (Hong et al., 2019). This finding does not imply that Sill is superior to these established measures; rather, it indicates that Sill provides complementary information by quantifying how functional similarity decays with spatial distance—a feature not directly captured by conventional univariate or connectivity-based metrics. We therefore interpret variogram-derived metrics as an additional lens for examining cortical organization, rather than a replacement for existing approaches. The observed elevation in Sill within association cortices is specific to our adolescent cohort; this pattern suggests a deviation in the spatial-functional organization unique to the adolescent phase of ASD, rather than a static trait across the lifespan.

Consistent with these findings, temporal and associative cortical regions have consistently been implicated in ASD across functional imaging studies (Redcay and Courchesne, 2008). These areas contribute to language and social-communicative processing, domains frequently affected in ASD (Pelphrey et al., 2011). Association cortices are characterized by extended developmental timelines, high metabolic demands, and increased inter-individual variability, factors that may contribute to their vulnerability in neurodevelopmental conditions (Buckner and Krienen, 2013). The localization of Sill alterations within these regions is therefore consistent with broader evidence suggesting that higher-order cortical systems represent key loci of inter-individual and spatial-functional variability in ASD. The observed association between Sill alterations and social-affective symptom severity further supports the behavioral relevance of spatial functional heterogeneity within these regions. Notably, we did not observe significant associations between any individual cluster and clinical symptom measures; the observed pattern reflects distributed, network-level effects rather than focal regional associations. No association was detected with restricted and repetitive behaviors, consistent with prior work suggesting partially dissociable neural correlates across ASD symptom domains (Abbott et al., 2016).

The significant positive association between elevated Sill (a metric of spatial-functional coupling heterogeneity) in higher-order association cortices and social-affective symptom severity provides preliminary evidence of a potential brain–behavior relationship, though the modest effect size (r≈0.30) warrants cautious interpretation. Neurobiologically, Sill quantifies the degree of spatial variability in resting-state functional connectivity; higher values reflect greater spatial heterogeneity in how functional signals are organized across the cortical surface. Potential links to local-to-global functional integration are discussed below as biologically plausible interpretations rather than direct properties measured by Sill. The identified Sill-altered regions—encompassing the default mode network (DMN), salience network, and fronto-temporal association cortices—are well-established as core hubs of social cognition, emotion processing, and interoceptive awareness in both neurotypical and ASD populations. Elevated Sill in these systems may reflect altered spatial organization of functional signals within regions known to support social cognition and affective processes. However, the correlational nature of our data precludes causal inferences about whether elevated Sill is directly linked to impaired neural coordination underlying these cognitive functions. We therefore interpret this association as a statistical link between spatial-functional heterogeneity and symptom severity, rather than evidence of mechanistic disruption. This finding aligns with prior work demonstrating that functional connectivity heterogeneity in association cortices is a hallmark of ASD pathophysiology, and extends these observations by linking this spatial heterogeneity pattern specifically to the severity of social-communicative impairments, the defining clinical feature of ASD.

Clinically, this association suggests a statistical link between spatial-functional heterogeneity and social-affective symptom severity in adolescent ASD (Di Martino et al., 2014). However, with an effect size of r≈0.30 explaining < 10% of the variance, this association is modest in magnitude and should be interpreted as preliminary evidence of a potential brain–behavior relationship rather than a definitive clinical substrate. Given the cross-sectional design and the absence of independent validation, these findings should be interpreted as preliminary associations rather than evidence of clinical utility. Independent replication in larger, well-phenotyped cohorts is necessary before drawing translational conclusions (Hull et al., 2017). Importantly, the specificity of the association to social-affective symptoms (rather than global severity alone) suggests that Sill may serve as a useful target for future mechanistic studies of social dysfunction in ASD, though this remains a hypothesis requiring independent replication (Hong et al., 2019).

Regarding age range consideration, in the present study, we restricted our analyses to participants aged 12–18 years. This age range was deliberately chosen based on neurodevelopmental evidence demonstrating that age 12 marks a critical transition from childhood to early adolescence, characterized by major changes in synaptic pruning, myelination, and large-scale network reorganization (Blakemore, 2012). Importantly, recent resting-state fMRI studies using the same ABIDE database have shown that children ( ≤ 12 years) and adolescents (12–18 years) exhibit qualitatively different connectivity patterns in ASD, with a developmental shift from hyper-connectivity in childhood to predominantly hypo-connectivity in adolescence (Shafieizadegan et al., 2025). This nonlinear trajectory suggests that including younger children would introduce fundamentally different neural signatures that could obscure adolescent-specific mechanisms. The upper bound of 18 years was adopted because by age 18–20, most subcortical white matter and association pathways reach a developmental plateau (Dayan et al., 2010), and cognitive control mechanisms in ASD differ qualitatively between adolescents and adults (Solomon et al., 2014). Moreover, the 12–18 age window has been widely used as a standard adolescent cohort definition in ASD neuroimaging research (Floris et al., 2021). Therefore, we believe that this age restriction, while limiting generalizability, is methodologically justified for isolating adolescent-specific distance-dependent functional organization in ASD.

With respect to sex-specific considerations, given the well-established role of sex as a modulator of adolescent brain development, we conducted supplementary sex-stratified analyses to examine whether the observed Sill differences between ASD and TD participants differed between males and females. As shown in Supplementary Figure 10, in the male subgroup, ASD participants showed elevated Sill values compared to TD participants within the primary difference regions reported in our main analysis. In the female subgroup, the same directional trend—higher Sill values in ASD relative to TD—was observed, but the difference did not reach statistical significance. We acknowledge this as a limitation of the present study. The lack of statistical significance in the female subgroup is likely attributable to the relatively small sample size of female participants in the ABIDE-I dataset, as efforts to unravel sex differences in the brain organization of autism have been challenged by the limited availability of female data (Floris et al., 2021). Consistent with this interpretation, a clear relation between cognition and brain activation or connectivity patterns across sexes is far from being established, and most findings remain exploratory (Taddei et al., 2023). We fully acknowledge that our sample size for female participants precluded a fully powered sex-stratified analysis, and therefore, our conclusions should be interpreted with caution when generalizing to females with ASD. Future studies with larger, sex-balanced samples—ideally with oversampling of female participants with ASD—are needed to systematically investigate potential sex differences in Sill and its neural correlates.

The absence of a significant association between Sill and RRB scores may be explained by several neurobiological and methodological factors. First, neurobiologically, social-affective and RRB symptoms in ASD are thought to arise from partially distinct neural systems: social deficits are primarily linked to higher-order association cortices (the focus of the current Sill analysis), while RRB symptoms are more strongly associated with subcortical structures (e.g., basal ganglia, thalamus), primary sensorimotor cortices, and cerebellar circuits, which were not the primary focus of our cortical Sill analysis (Bertelsen et al., 2021). Our variogram-derived metrics were computed exclusively on the cortical surface, and thus did not capture the spatial-functional coupling abnormalities in subcortical and sensorimotor systems that are implicated in RRB (Hansen et al., 2022).

Methodologically, the adolescent ASD sample in this study may have limited variability in RRB severity, or RRB symptoms may be more strongly modulated by factors not fully accounted for in the current analysis (e.g., comorbid psychopathology, psychotropic medication use, or developmental stage). Additionally, RRB symptoms are heterogeneous, encompassing repetitive motor behaviors, sensory sensitivities, and insistence on sameness, and the ADOS RRB domain may not fully capture the full spectrum of these behaviors in adolescent populations. Finally, it is possible that Sill, as a potential index of cortical association network integration, is specifically tuned to social-cognitive deficits, and does not index the distinct neurobiological mechanisms underlying RRB. Future work will extend the Sill analysis to subcortical and sensorimotor regions, and incorporate more granular RRB phenotyping, to further investigate this relationship.

Network-level analyses indicated that Sill alterations were concentrated within higher-order functional systems, particularly the language network and multimodal association networks (including the default mode and frontoparietal networks). Previous studies have shown that association networks exhibit prolonged developmental trajectories and increased susceptibility to neurodevelopmental variation (Sydnor et al., 2021). The absence of significant effects in primary sensory networks suggests that ASD-related spatial alterations may preferentially affect integrative cortical systems rather than early sensory processing regions. It is worth noting that while vertex-wise analyses revealed alterations in motor regions, these effects did not survive network-level averaging. This discrepancy may be attributed to the relatively large size of primary sensory/motor networks compared to higher-order networks, where averaging across heterogeneous vertices may dilute focal spatial alterations. Therefore, the absence of significant effects in primary sensory networks at the network-averaged level does not preclude focal alterations at the vertex level, but suggests that the overall integrative function of these networks may be less affected compared to the Language and Multimodal networks.

To contextualize the variogram-derived Sill within the broader landscape of cortical spatial organization, we examined its relationship with previously reported spatial metrics. Leech et al. (2023) demonstrated that the Sill—defined as the asymptotic variance of the fitted variogram—varies systematically along the principal gradient of functional connectivity, a low-dimensional embedding that recapitulates the sensory-transmodal cortical hierarchy (Leech et al., 2023). Specifically, regions closer to the transmodal end of this gradient (e.g., default mode and frontoparietal networks) exhibit higher Sill values, reflecting more rapid functional changes over short cortical distances, whereas regions at the unimodal end (e.g., visual and motor cortices) exhibit lower Sill values, reflecting more gradual functional changes with distance. Importantly, while this gradient-based framework captures a continuous axis of cortical organization, the Sill provides a complementary, vertex-wise quantification of how functional similarity decays with spatial distance within each local cortical neighborhood. Moreover, Leech et al. (2023) further showed that the spatial distribution of Sill is associated with intracortical myelin content, with more heavily myelinated regions displaying longer spatial dependencies and lower Sill values, suggesting a link between spatial-functional heterogeneity and underlying microstructural properties. Together, these prior findings establish that Sill captures a related but distinct aspect of cortical organization—one that reflects the spatial scale and rate of functional differentiation across the cortical mantle. We therefore do not claim that Sill replaces these established metrics; rather, we argue that Sill provides a spatially explicit, parameterized description of functional similarity decay that complements gradient-based and autocorrelation-based approaches.

Functional decoding analyses demonstrated overlap with cognitive domains including language, working memory, and social cognition. These findings align with meta-analytic evidence linking ASD to atypical recruitment of distributed cognitive systems rather than isolated functional deficits (Yeo et al., 2011). Importantly, these results describe statistical correspondence rather than mechanistic specificity.

Integration with neurotransmitter reference maps revealed that multiple neuromodulatory systems jointly accounted for a portion of the spatial variance in ASD-related Sill differences. Neuromodulatory systems such as acetylcholine and norepinephrine are known to influence large-scale cortical dynamics and signal gain regulation (Shine et al., 2019). However, we emphasize that these associations are correlational and do not establish mechanistic links. The observed associations therefore suggest that spatial functional variability may relate to distributed neurochemical organization, although causal interpretations cannot be inferred. While the present results do not establish mechanistic links, the observed correspondence supports the hypothesis that spatial-functional organization may be statistically constrained by underlying neurochemical architecture (Deco et al., 2018).

Transcriptomic analyses further demonstrated a statistical spatial correspondence between ASD-related Sill alterations and cortical gene expression patterns, offering a reference-based framework for contextualizing the observed spatial-functional organization (Voineagu et al., 2011). Converging evidence from prior transcriptomic and genetic studies has consistently implicated synaptic dysfunction as a central feature of ASD (Parikshak et al., 2013), with risk genes enriched for processes related to synaptic signaling, neuronal communication, and activity-dependent plasticity. In addition to neuronal mechanisms, non-neuronal contributions have been increasingly recognized, with glial and immune-related processes—particularly involving microglia and astrocytes—showing altered gene expression profiles in ASD (Velmeshev et al., 2019). Mitochondrial dysfunction has also been linked to ASD, potentially reflecting altered cellular energy metabolism and increased vulnerability of metabolically demanding cortical systems during neurodevelopment.

Within this spatial correspondence framework, the enrichment of genes related to synaptic signaling, mitochondrial processes, and glial function observed in the present study aligns closely with established molecular signatures of ASD in. Notably, these transcriptional features are not uniformly distributed across the cortex but are preferentially expressed in higher-order association regions, which are characterized by prolonged developmental trajectories, elevated metabolic demands, and increased transcriptional diversity. Importantly, because our imaging–transcriptomic analyses establish spatial correspondence rather than biological mediation, these findings should be interpreted as statistical alignment between independently derived imaging and transcriptomic reference maps rather than as evidence that the observed spatial patterns are causally linked to underlying molecular processes in our cohort. Although the AHBA data provide a valuable reference framework, the gene expression data were derived from adult neurotypical donors, whereas our study cohort consists of adolescents with ASD. Future studies using developmentally matched and disorder-relevant transcriptomic data would be needed to examine whether these spatial patterns reflect underlying biological susceptibility. Prior imaging-transcriptomic work has similarly demonstrated that macroscale functional organization is constrained by spatial gradients of gene expression, particularly those related to synaptic and metabolic processes (Burt et al., 2020), providing further support for a multiscale correspondence between functional architecture and underlying molecular systems (Fornito et al., 2019).

The disease enrichment results further extend this interpretation by situating the identified gene sets within the broader landscape of neurodevelopmental and psychiatric risk (Grove et al., 2019). Specifically, the observed enrichment for intellectual disability and schizophrenia-related gene sets is consistent with large-scale genomic studies demonstrating substantial overlap between ASD risk genes and those implicated in other neurodevelopmental and psychiatric conditions (Lee et al., 2019). This observation is consistent with large-scale genomic studies reporting shared genetic architectures across diagnostic boundaries. However, because our analysis is based on spatial correspondence between imaging-derived patterns and reference gene sets rather than direct genetic assays in the study cohort, these findings should be interpreted as statistical alignment with prior genomic knowledge rather than evidence of shared etiological mechanisms in our sample (Hong et al., 2019), consistent with our interpretation of these analyses as establishing spatial correspondence rather than biological mediation.

Taken together, the overlap between imaging-derived spatial-functional alterations and transcriptional signatures enriched for ASD-relevant and cross-disorder risk genes supports a multiscale interpretation in which macroscale cortical organization is statistically aligned with molecular reference profiles derived from independent datasets. This alignment does not establish that the observed spatial patterns reflect underlying molecular vulnerability in our cohort, but rather provides a statistical framework for generating hypotheses about potential biological correlates.

In contrast to the widespread differences observed for Sill, the Range parameter showed no significant group differences. To quantify the evidence supporting this null finding, we performed vertex-wise Bayesian analysis (Supplementary Table 6). The results showed that 73% of vertices had BF_01_ > 0.33, indicating that they do not provide strong evidence for a difference, with a whole-brain median BF_01_ of 0.83, suggesting limited overall evidence for systematic cross-vertex differences. These findings statistically support the conclusion that the absence of Range effects is unlikely to be driven by insufficient power. From a biological perspective, Sill and Range reflect different aspects of cortical spatial organization. Sill represents the asymptote of the variogram, essentially capturing the overall strength of spatial dependence between a given vertex and all other vertices across the whole brain— a comprehensive measure of how local morphological features covary with the rest of the cortex. The diffuse morphological alterations widely reported in ASD, such as changes in cortical thickness and surface area, are likely to affect this overall covariance strength first. In contrast, Range reflects the characteristic distance over which spatial dependence decays—that is, the distance over which cortical morphological features maintain similarity with one another—capturing the local topological organization of cortical space. Importantly, the spatial covariance patterns of cortical morphology are largely established early in development and are strongly constrained by genetic factors, representing fundamental principles of cortical spatial organization. The pathophysiological processes underlying ASD may primarily affect the *expression* of morphological features (i.e., the magnitude of thickness or area) rather than the fundamental decay patterns of spatial correlations between cortical regions. Thus, when a disease predominantly alters the amplitude rather than the spatial organizational mode of morphological features, Sill shows differences while Range remains unchanged—a pattern that is biologically plausible.

Several limitations should be considered.

First, the cross-sectional design precludes inference regarding developmental trajectories. Although we reference developmental patterns from prior literature, we acknowledge that our data cannot capture longitudinal changes or true developmental processes, and claims about “development” should be interpreted with caution.

Second, multisite data introduce potential variability despite statistical harmonization procedures (Fortin et al., 2018).

Third, transcriptomic and neurotransmitter reference datasets are derived from independent samples and therefore represent indirect correspondence rather than subject-specific biological measurements. Specifically, the Allen Human Brain Atlas (AHBA) has several constraints: (1) the donors were neurotypical adults, whereas our study cohort consists of individuals with ASD who are predominantly adolescents; (2) the small sample size (*n* = 6 donors) limits statistical power; (3) the data are from post-mortem tissue, making the link to in-vivo functional imaging indirect. Furthermore, given that gene expression is highly dynamic during neurodevelopment, the adult AHBA data may only provide a coarse approximation of the spatial transcriptomic patterns relevant to adolescent brain organization. Therefore, the observed correspondence should be interpreted as a static reference rather than a dynamic developmental mechanism. These factors limit the generalizability of the gene-imaging associations and should be considered when interpreting our transcriptomic findings. Fourth, spatial association analyses quantify statistical alignment and should not be interpreted as evidence of causal biological mechanisms. Finally, our imaging metrics (Sill and Range) reflect neural signal variability patterns but cannot directly infer underlying neurochemical mechanisms. Claims about specific neurotransmitter systems (e.g., cholinergic, noradrenergic, or serotonergic) would require direct evidence from molecular imaging or pharmacological studies. Future multimodal investigations combining the current approach with PET imaging or other neurochemical assays would be needed to examine neurotransmitter-specific contributions to the observed network alterations. Similarly, these findings should not be interpreted as evidence for clinical biomarker utility.

Forth, Detailed medication data were available for only a subset of participants, and medication profiles within the ASD group were highly heterogeneous—including the use of SSRIs, stimulants, and antipsychotics, among others. Given this heterogeneity and the limited sample size, we were unable to perform stratified subgroup analyses by medication class or incorporate medication dose and type as covariates in our statistical models. Consequently, we cannot rule out the possibility that medication effects may partially contribute to the observed group differences. It should also be noted that the inclusion of medication status as a covariate in case–control comparisons is methodologically challenging: healthy controls lack medication information, which would introduce missing data, and even if a value of zero were artificially assigned, this variable would be highly collinear with the diagnostic grouping variable, rendering the statistical model unable to disentangle group effects from medication effects. Future studies with comprehensively phenotyped cohorts, including detailed medication histories, will be essential to replicate our findings and to clarify the extent to which pharmacotherapy may influence the reported neurotransmitter–connectivity associations.

Fifth, we acknowledge that our restriction to participants aged 12–18 years limits the generalizability of our findings. However, we would like to briefly clarify the rationale behind this choice. This age range was deliberately selected because age 12 marks a critical transition from childhood to early adolescence, characterized by major changes in synaptic pruning, myelination, and large-scale network reorganization (Blakemore, 2012). Recent resting-state fMRI studies using the same ABIDE database have shown that children ( ≤ 12 years) and adolescents (12–18 years) exhibit qualitatively different connectivity patterns in ASD, with a developmental shift from hyper-connectivity in childhood to predominantly hypo-connectivity in adolescence (Shafieizadegan et al., 2025). The upper bound of 18 years was adopted because by age 18–20, most subcortical white matter and association pathways reach a developmental plateau (Dayan et al., 2010), and cognitive control mechanisms in ASD differ qualitatively between adolescents and adults (Solomon et al., 2014). Therefore, while this age restriction limits generalizability to younger children and older adults, we believe it is methodologically justified for isolating adolescent-specific distance-dependent functional organization in ASD. Nevertheless, we recognize several limitations associated with this choice. The World Health Organization defines adolescence as ages 10–19 years, and our narrower age range does not capture the full spectrum of adolescent development. Younger adolescents (ages 10–11) and older adolescents (ages 18–19) may exhibit different patterns of distance-dependent functional organization that we were unable to examine. Moreover, developmental heterogeneity within the 12–18 range remains substantial. Pubertal timing varies considerably across individuals of the same chronological age (Tanner, 1986), and we did not have direct measures of pubertal status in the ABIDE-I dataset. Consequently, our conclusions should be interpreted as primarily applicable to adolescents within the 12–18 age range. Future studies incorporating broader age ranges with appropriate statistical controls for nonlinear developmental trajectories, as well as direct measures of pubertal status, are needed to determine the generalizability of the current findings across the entire lifespan.

Sixth, the ABIDE-I dataset does not contain standardized measures of educational attainment. As documented in the official release paper (Di Martino et al., 2014), educational level was not uniformly collected across the 17 contributing sites. This limitation is not unique to our study; other researchers using ABIDE-I have faced the same constraint (Heinsfeld et al., 2018). Moreover, systematic reviews have reported that demographic variables—including socioeconomic indicators—are inconsistently reported in neuroimaging research, with only 19.4% of studies reporting socioeconomic makeup (Gard et al., 2025) and fewer than 4% reporting race and ethnicity information (Oyeniran et al., 2025), largely because public datasets often lack this information. To partially address this concern, we used IQ as an indirect proxy for educational level. Meta-analytic evidence confirms a positive correlation between IQ and education, both in general populations (Ritchie and Tucker-Drob, 2018) and specifically in ASD (Marinopoulou et al., 2026). As shown in our Supplementary Figure 6, after multiple comparison correction, no clusters overlapped with our primary ASD–TD Sill difference maps, suggesting that our core findings are unlikely to be substantially confounded by educational level. Nevertheless, future studies with standardized educational assessments are needed.

Seventh, methodological limitations should be noted. Regarding motion control, we employed an FD threshold of <0.3 mm for volume censoring, consistent with the ABIDE standard preprocessing pipeline. While more stringent thresholds (e.g., 0.2 mm) have been proposed for resting-state analyses, we chose 0.3 mm to balance artifact removal with data retention— particularly important given the higher motion burden reported in ASD cohorts. We included mean FD as a covariate and confirmed that motion did not significantly correlate with our variogram parameters. However, we did not systematically test alternative motion thresholds, which constitutes a limitation. Regarding parcellation, we used the Schaefer 100-parcel atlas (Schaefer et al., 2018), which has demonstrated good reliability and neurobiological validity, but remains a group-average template that may not fully capture inter-individual variability. Although our vertex-level analytical framework inherently reduces dependence on any specific parcellation scheme, we did not systematically compare alternative parcellations, and this remains a limitation. Future studies may examine the robustness of these findings across a wider range of motion correction strategies and parcellation schemes.

## Materials and methods

### Participants

#### Materials/measurements

##### Ethics statement

This study was conducted in accordance with the Declaration of Helsinki. All data were obtained from the publicly available Autism Brain Imaging Data Exchange (ABIDE-I) repository. Each participating institution obtained approval from its local institutional review board, and written informed consent was obtained from all participants or their legal guardians prior to data acquisition. The present study only performed secondary analyses of anonymized, de-identified imaging and phenotypic data; therefore, additional ethical approval was not required. Data usage strictly complied with the ABIDE data-sharing protocols and terms of use.

##### Human subjects and MRI data acquisition

In our study, a public dataset from the ABIDE-I was used to reveal aberrant Range and Sill in adolescent ASD. We analyzed a well-characterized subset of data from the Autism Brain Imaging Data Exchange (ABIDE) repository, focusing on adolescents aged 12–18 years with high-quality resting-state fMRI data and complete clinical assessments. This subset was selected to ensure phenotypic homogeneity (age-matched cohorts, minimal motion artifacts, and consistent Autism Diagnostic Observation Schedule calibrated severity scores, which quantify autism symptom severity), a critical consideration for investigating subtle neurodevelopmental differences in spatial-functional network organization (Gotham et al., 2009). Data selection followed standardized criteria outlined in the ABIDE-I data use protocol,^1^ with additional quality control to exclude scans with excessive head motion ( > 2 mm translation/2° rotation) or incomplete phenotypic information. Detailed demographic information for the ASD group is provided in ASD_Information.xlsx, while the demographic data for the TD group is provided in TD_Information.xlsx.

This study used publicly available human neuroimaging data from the ABIDE-I. All data were originally collected with approval from local institutional review boards at each participating site, and written informed consent was obtained from all participants or their legal guardians. The present study involved secondary analysis of de-identified data and did not require additional ethical approval. Data usage complied with the ABIDE data sharing agreement.

Participants were recruited from multiple imaging sites. To minimize potential site-related confounds, site was included as a covariate in all group-level statistical analyses.

Inclusion criteria:

1. Participants were adolescents aged 12–18 years from the ABIDE-I database.
2. Participants were diagnosed with ASD or TD using standardized assessments, including the ADOS and the Autism Diagnostic Interview-Revised (ADI-R).
3. Each imaging site included at least 10 participants in total (ASD + TD combined).
4. Participants had valid high-quality rs-fMRI data and complete basic demographic information.

Exclusion criteria:

1. Sites with fewer than 10 total participants (ASD + TD) were excluded to avoid unstable site effects.
2. Participants with excessive head motion (mean FD ≥ 0.3 mm) were excluded to mitigate motion-related artifacts, a threshold commonly adopted in the literature to ensure data quality (Abraham et al., 2017).
3. Participants with missing key demographic or clinical information.

Psychotropic medication use:

All resting-state fMRI data were acquired across 10 imaging sites with site-specific repetition times (TR):

- 1,500 ms (OLIN, PITT);
- 1,667 ms (LEUVEN_2);
- 2,000 ms (NYU, SDSU, TRINITY, UM_1, UM_2, USM);
- 3,000 ms (UCLA_1).

Scan durations varied across sites, ranging from 5.06 to 8.06 min. Site-specific TR values and sample sizes are further detailed in Supplementary Table 7.

Detailed demographic characteristics, including age, sex, handedness, IQ, head motion (framewise displacement), MRI acquisition parameters, and group-comparison statistics for ASD and TD participants within each acquisition site are summarized in Supplementary Table 7. We conducted a sensitivity analysis on the data after applying GSR preprocessing, and the results also supported the current conclusion (Supplementary Figure 11).

##### Human fMRI data preprocessing

Functional MRI data processing was conducted using the fMRIPrep 22.1.1 pipeline (Esteban et al., 2019) as the foundational framework, with integration of the Nipype neuroimaging processing library for flexible workflow construction and management (Gorgolewski et al., 2011). Structural MRI preprocessing included essential steps of intensity normalization, brain extraction, tissue segmentation, cortical surface reconstruction, and spatial normalization to the standard template space, while functional MRI preprocessing incorporated head motion correction, slice-timing (inter-slice time) correction, and rigid-body registration to structural images. Furthermore, a “simple” confound removal strategy implemented in the Nilearn package was applied to reduce spurious variance in functional signals, including high-pass filtering, regression of motion-related signals and tissue signals derived from white matter and cerebrospinal fluid, signal detrending, and subsequent z-score standardization (Lindquist et al., 2019). The definitions of the geodesic distance matrix, functional connectivity matrix and fitted variogram are illustrated through the following calculation process.

(1) The Conte32k template served as the foundational anatomical space. After cortical surface reconstruction and inflation, an exclusion mask was applied to discard non-cortical and irrelevant vertices, yielding two hemisphere-specific vertex sets—29,696 for the left and 29,716 for the right hemisphere, totaling 59,412 valid vertices across the whole cortex. Critically, these vertices were not treated as independent observations but as nodes embedded in a continuous two-dimensional manifold. To quantify their spatial embedding, we employed the BrainSMASH toolbox (Burt et al., 2020), which—although originally devised for generating spatially autocorrelated surrogate maps—provided the necessary infrastructure for computing inter-vertex geodesic distances along the cortical surface. The output was a full pairwise distance matrix encompassing all 59,412 vertices, which was subsequently frozen as the immutable spatial coordinate system anchoring every downstream analysis.

(2) This track operated at the level of individual participants, drawing entirely on resting-state BOLD signal fluctuations. For each participant, we computed a vertex-wise Pearson correlation matrix (59,412 × 59,412), capturing the temporal synchrony between every possible pair of cortical vertices. To render these correlation coefficients amenable to subsequent averaging and statistical operations, each coefficient was individually converted to Fisher’s z-score, thereby mitigating the bounded nature and skewness of the correlation distribution. These transformed values were then averaged across participants on a vertex-by-vertex basis. Finally, the pooled z-statistics were back-transformed to the correlation scale via the inverse Fisher-z transformation, yielding a group-level functional connectivity matrix whose entries naturally fell within the closed interval [-1, 1]. In this matrix, a value of zero signifies the absence of any linear relationship, while values approaching the extremes denote near-perfect monotonic dependencies. Importantly, this bounded and population-averaged metric ensured that connectivity estimates were directly comparable across vertices and individuals—a property indispensable for the stable estimation of spatial variograms in the subsequent modeling step.

(3) The variogram measures how functional similarity between cortical regions decays as a function of geodesic distance. This relationship is summarized by two parameters: the Sill, defined as the asymptotic variance parameter of the fitted variogram model, which quantifies the maximum functional heterogeneity across the cortex by capturing the extent to which functional similarity decreases with increasing spatial distance; and the Range, defined as the distance parameter at which this asymptotic variance is reached, which quantifies the spatial scale over which functional similarity between cortical regions persists. Both parameters are statistical descriptors of spatial-functional organization rather than direct measures of neural integration or connectivity; their biological interpretations are discussed as plausible inferences derived from the spatial statistics literature.

To systematically characterize cortical functional connectivity, we adopted a geostatistical framework modeling functional similarity between vertices as a function of geodesic distance. The empirical variogram served as our primary tool, quantifying how signal dissimilarity increases with cortical distance. At the core of this framework lies a dissimilarity metric tailored to correlation-based connectivity: for each vertex pair, we defined *D* = 1 – |ρ|, where ρ is the Pearson correlation between BOLD time series. This index ranges from 0 (perfect synchrony) to 1 (no correlation), reframing functional connectivity as a distance-dependent decay process. We partitioned the full geodesic distance spectrum into 20 evenly spaced bins and computed the average dissimilarity per bin, yielding an empirical curve tracing the loss of functional coupling with anatomical separation. We fitted this empirical relationship to an exponential model via nonlinear least squares, restricting the fitting to bins 2–19 to exclude sparsely populated extremes and truncating the distance axis at 150 mm. This yielded two interpretable coefficients: the Sill—the asymptotic variance capturing maximal functional divergence across the cortex—and the Range—the distance at which this asymptote is reached, indexing the spatial persistence of functional coupling.

Beyond global characterization, we extended this logic to the vertex level via row-wise variogram estimation for each vertex, generating spatially explicit parametric maps (Sill and Range) that reveal regional variations obscured in global aggregates. All parameter estimation for both global and vertex-wise models followed the protocols of Leech et al. (2023).

##### Vertex-wise group comparison

We performed vertex-wise comparisons of Sill and Range between the ASD and TD groups across the entire cortical surface. At each cortical vertex, Range and Sill values were compared using two-sample independent *t*-tests (two-tailed), generating a T-statistic map representing group differences. Range and Sill difference scores were statistically compared between ASD and TD using surface-based linear models implemented in SurfStat^2^ for Matlab. Models included effects of age, sex, site, mean FD and group. Surface-based findings were corrected for family-wise errors due to multiple comparisons using a random field theory of *P*__*FWE*_ < 0.05.

##### Symptom-imaging association analysis

To examine the association between imaging-derived Sill measures and symptom severity in ASD, we performed partial correlation analyses within the ASD group of the ABIDE-I dataset, as Autism Diagnostic Observation Schedule (ADOS) scores were available for all participants in this cohort. Symptom severity was indexed using the ADOS total score and its subscales, including social affect, restricted and repetitive behaviors (RRB), and calibrated severity scores where applicable.

##### Network-level aggregation

To examine network-specific patterns, we mapped vertex-wise Sill values onto a 12-network functional parcellation of the cerebral cortex. Each cortical vertex was assigned to one of the 12 canonical functional networks according to the atlas definition.

For each participant, mean Sill values were calculated within each network by averaging across all vertices belonging to that network. This procedure yielded 12 network-level Sill measures per individual, which were used for subsequent statistical analyses.

##### Meta-analytic cognitive decoding

Observed neural signatures are projected onto cognitive feature spaces via a reverse-inference engine powered by large-scale meta-analytic resources. To decode the cognitive relevance of our cortical findings, a reverse-inference pipeline was implemented. Raw Neurosynth correlation outputs were Fisher-transformed (*z* > 3.1, FDR *p* <0.05), projected onto cortical vertices, and binned into 20 intensity intervals. The underlying search space comprised 24 behavioral and cognitive terms (Margulies et al., 2016; Preti and Van De Ville, 2019), spanning social, executive, language, and perceptual processing. All meta-analytic maps were retrieved from Neurosynth.^3^

Building upon these Neurosynth-derived topographies, we integrated receptor/transporter density maps from > 1,200 healthy individuals (Hansen et al., 2022). After sample-size weighting, nine neurotransmitter system-level maps (acetylcholine, cannabinoid, dopamine, GABA, glutamate, histamine, norepinephrine, opioid, serotonin) were resampled onto the Schaefer 100 atlas. Using JuSpace (A toolbox that tests whether two brain maps are spatially colocalized by determining if their patterns overlap more than expected by chance while accounting for spatial autocorrelation.), we analyzed single-receptor and class-level associations with Sill differences (Dukart et al., 2021). A multiple linear regression quantified each system’s contribution, with robustness assessed via bootstrap and permutation testing (10,000 each). To clarify the statistical framework of our neurotransmitter association analysis, we have added the explicit multiple linear regression equation in the Methods section, which models the spatial association between the ASD-TD Sill difference map (denoted as Ov at cortical vertex v) and the densities of 9 key neurotransmitter systems:


$$O_{v}=\beta_{0}+\beta_{1}\times \text{Acetylcholine}+\beta_{2}\times \text{Cannabinoid}+$$

$$\beta_{3}\times \text{Dopamine}+\beta_{4}\times \text{GABA}+\beta_{5}\times \text{Glutamate}$$

$$+\beta_{6}\times \text{Histamine}+\beta_{7}\times \text{Norepinephrine}$$

$$+\beta_{8}\times \text{Opioid}+\beta_{9}\times \text{Serotonin}+\epsilon_{v}$$

Where:

• *Ov* = ASD-TD Sill difference value at cortical vertex v;

• Acetylcholine/Cannabinoid/Dopamine/GABA/Glutamate/ Histamine/Norepinephrine/Opioid/Serotonin = Regional density of each neurotransmitter receptor system at vertex v;

• *β_0_* = Intercept; *β_1_–β_9_* = Regression coefficients for each neurotransmitter system;

• *ϵ_*v*_* = Residual error term.

##### Ridge regression analysis

To investigate the contribution of neurotransmitter systems to the observed spatial pattern of ASD-related alterations while minimizing the influence of multicollinearity among neurotransmitter maps, we performed ridge regression analyses. Prior to model fitting, all neurotransmitter receptor density maps were standardized to have zero mean and unit variance. Ridge regression models were then fitted using the cortical alteration map as the dependent variable and nine neurotransmitter system maps as predictors. The optimal regularization parameter (α) was determined using five-fold cross-validation over a logarithmically spaced range of values (10^-4 to 10^4), selecting the parameter that minimized the cross-validated mean squared error. Regression coefficients obtained under the optimal α were used to quantify the relative contribution of each neurotransmitter system. To evaluate the stability of model coefficients, a bootstrap procedure with 5,000 resamples was performed. For each bootstrap iteration, cortical regions were resampled with replacement, the ridge regression model was refitted using the optimal α, and regression coefficients were recorded. The resulting coefficient distributions were used to estimate bootstrap confidence intervals and assess coefficient stability. To further evaluate statistical significance, permutation testing was conducted using 10,000 permutations of the dependent variable while preserving the predictor structure. For each permutation, the ridge regression model was refitted using the same regularization parameter, generating a null distribution of regression coefficients. Two-tailed empirical *p*-values were calculated as the proportion of permuted coefficients whose absolute values exceeded the observed coefficient magnitude. Statistical significance was determined using permutation-derived *p*-values, with correction for multiple comparisons performed using the false discovery rate (FDR) procedure where appropriate.

##### Partial least squares regression

To elucidate molecular mechanisms, we integrated transcriptomic data from the Allen Human Brain Atlas.^4^ Microarray expression data from six postmortem brains were processed using the Abagen toolbox (Arnatkeviciute et al., 2019; Markello et al., 2021) and mapped to the Schaefer 100 atlas (Markello et al., 2021). Partial least squares (PLS) regression was employed with gene expression as predictors and Sill differences as responses. A multivariate technique that identifies latent components maximizing the covariance between two high-dimensional datasets. Permutation testing assessed spatial autocorrelation (10,000 rotations). Bootstrapping estimated gene weights (Morgan et al., 2019). Significant PLS1 genes (FDR < 0.05) underwent enrichment analysis via Metascape (Zhou et al., 2019) for GO biological processes. Laminar enrichment analyses were performed using published marker gene lists (Maynard et al., 2021). Cell-type-specific gene lists were assembled from a prior study (Seidlitz et al., 2020). The aggregate fold change method was used for enrichment analyses (Pirooznia et al., 2012), with FDR correction applied.

##### Single-cell data processing and cell-type assignment

The Lake data are publicly available from the Gene Expression Omnibus (GSE97942). We used identical processing pipelines for the single-cell datasets with the Seurat toolkit (v3), consistent with those applied by Anderson (Anderson et al., 2020). First, initial filtering was conducted to remove genes expressed in less than three cells and exclude cells with fewer than 200 expressed genes. Then log normalization (“NormalizeData” function) was performed for gene expressions of each cell, based on total expression values, using a size factor of 10,000 molecules. Finally, we regressed out covariates of the sequencing platform and batch processing, and the residuals were scaled and centered (“ScaleData” function). We used predefined cell classes from each single-cell dataset and identified transcriptional markers (that is, differentially expressed genes) for each cell type based on the Wilcoxon rank-sum test (“FindMarkers” function). We identified 16 cell classes in the Lake dataset according to previously established annotations (Anderson et al., 2020), encompassing cell types defined in both the frontal cortex and Visual Network (VIS), including five interneurons (In1, In3, In4, In6 and SST), five excitatory neurons (Ex1, Ex3, Ex4, Ex5 and Ex8) and six non-neuronal cells (astrocyte (Ast), oligodendrocyte (Oli), pericyte (Per), endothelial (End), microglia (Mic) and oligodendrocyte precursor cell (OPC)).

##### Identification of Sill t-map-related cell types by cell enrichment analysis

We identified cell-type-specific genes, according to positive and significant differential expressions, that survived the Bonferroni correction at the level of 0.05. The positive value indicated a high level of expression of the respective gene in that particular cell type. For the Lake cells, these analyses were accomplished separately for the frontal cortex and VIS samples, and differentially expressed genes were intersected. We performed cell enrichment analysis for abnormal Sill-associated genes obtained from the imaging transcriptomic analysis, using the Fast Gene Set Enrichment Analysis (FGSEA) method (Korotkevich et al., 2016), which can quickly estimate arbitrarily low Gene Set Enrichment Analysis (GSEA) *P*-values with high accuracy. The Normalized Enrichment Score (NES), indicating the enrichment score normalized to the mean enrichment of random genes with the same size in each cell, and the significance level (FDR adjusted P) were given in this analysis. The enrichment score (ES) was calculated using the same running-sum statistic as implemented in the Broad Institute Gene Set Enrichment Analysis (GSEA) algorithm (Subramanian et al., 2005). In particular, we reported sign (NES) × -log_10_ (adjusted P) values, which indicated either a positive or negative enrichment, as well as the significance, simultaneously. If NES was positive, then sign (NES) was equal to one, otherwise to minus one.

##### Gene ontology enrichment analysis

A set of positively associated genes corrected with p <0.05 using the FDR method were selected for gene enrichment analysis using Metascape to identify significant term.

##### Statistical analysis

Details of the statistical methods, and the software and packages used in each relevant section have been described above, with all remaining analyses performed using Python v3.7. Before conducting any statistical analysis, participants with incomplete essential data or failing quality control, as well as those who did not meet the inclusion criteria, were excluded (see the “Participants” section). For the permutation analyses used in the transcriptomic work, we employed a standard spin permutation approach (Alexander-Bloch et al., 2018) to account for spatial autocorrelation in the brain surface data. A permutation approach that randomly rotates cortical maps on a spherical surface to create null distributions, thereby breaking inter-map associations while preserving each map’s intrinsic spatial structure. Specifically, this is a rotational permutation method as follows: To assess statistical significance, we applied spatial permutation null models with FDR correction (*p* <0.05). Each PLS1 score map was projected onto the fsLR32k surface space to create surface-based parcel representations. Spatial coordinates for each parcel were defined by selecting the vertex closest to the parcel centroid via spherical projection on the average surface. These parcel coordinates were then randomly rotated, and the original parcel values were reassigned to the nearest rotated parcel (10,000 iterations). Each random rotation redistributed surface parcel values across brain regions to construct a null distribution. The significance was determined using thresholds of *P*__*spin*_ < 0.05.

##### Multiple-comparison correction strategies

Different correction approaches were selected based on the specific characteristics of each analysis, following established practices in neuroimaging:

(1) Vertex-wise group comparisons (Sill and Range). We employed family-wise error (FWE) correction using random field theory (RFT) implemented in SurfStat. RFT is particularly suitable for smooth, continuous surface-based data because it accounts for spatial correlation among neighboring vertices and provides stringent control of family-wise error rates, consistent with established surface-based neuroimaging methods.

(2) Network-level and clinical association analyses. For comparisons across the 12 functional networks and for partial correlations between Sill and symptom measures, we applied FDR correction (Benjamini-Hochberg procedure). FDR correction is appropriate for moderate numbers of comparisons where the tests are not fully independent, as it controls the expected proportion of false positives while maintaining greater statistical power than FWE.

(3) Spatial correlation analyses (e.g., neurotransmitter and transcriptomic mappings). For spatial correlations between maps (e.g., ASD-TD Sill T-map vs. neurotransmitter densities; ASD-TD Sill T-map vs. gene expression), we used spin permutation testing (10,000 rotations) to preserve the spatial autocorrelation structure of cortical data. Significance was determined as *P_spin* < 0.05.

(4) Functional decoding (Neurosynth). For the meta-analytic term mapping (Figure 3C), we applied a conservative z-threshold (z > 3.1, equivalent to *p* <0.05, FDR-corrected across terms) as recommended by standard Neurosynth decoding practices.

(5) Exploratory visualizations. For descriptive heatmaps and exploratory figures (e.g., Figure 3C, the full correlation matrix), no formal multiple-comparison correction was applied; these are explicitly labeled as uncorrected and interpreted as hypothesis-generating rather than confirmatory.

## Data Availability

The original contributions presented in this study are included in the article/Supplementary material, further inquiries can be directed to the corresponding author.

## Footnotes

1
http://fcon_1000.projects.nitrc.org/indi/abide/

2
http://www.math.mcgill.ca/keith/surfstat/

3
https://neurosynth.org/

4
https://human.brain-map.org/
