# Supplementary material for: Exploring atypical spatial-functional coupling in adolescent autism spectrum disorder: insights from neurodevelopment and transcriptomic architecture
Source: Front Neurosci. 2026 Jul 27;20:1780430. doi: 10.3389/fnins.2026.1780430 (PMC13454100; doi:10.3389/fnins.2026.1780430)
Supplement: Supplementary file 1 [file Supplementary_file_1.pdf]

Region	ASD (Mean±SD)	TD (Mean±SD)	T	P	Cohen's d
1	0.48±0.04	0.45±0.03	4.85	1.88e-06	0.53
2	0.50±0.04	0.48±0.03	4.58	6.69e-06	0.50
3	0.48±0.04	0.46±0.03	5.28	2.35e-07	0.58

Supplementary Table 1. Effect sizes for ASD–TD group differences in Sill across significant clusters.

ADOS	Spearman's ρ	95% CI	P	P_{FDR}
ADOS_GOTHAM_SOCAFFECT	0.30	[0.08, 0.48]	0.009	0.037
ADOS_GOTHAM_RRB	-0.06	[-0.29,0.17]	0.611	0.611
ADOS_GOTHAM_TOTAL	0.25	[0.03, 0.44]	0.027	0.051
ADOS_GOTHAM_SEVERITY	0.23	[0.01, 0.43]	0.039	0.051

Supplementary Table 2. Correlations between Sill differences and ADOS symptom measures in the ASD group.

Neurotransmitter	coef	Std err	95% CI	t	P	P_{FDR}	Contribution
Acetylcholine	-0.5660	0.276	[-0.115, -0.017]	-2.048	0.043	0.122	22.6%
Cannabinoid	-0.1613	0.137	[-0.434, 0.111]	-1.175	0.243	0.312	6.4%
Dopamine	-0.0588	0.141	[-0.339, 0.222]	-0.416	0.678	0.714	2.3%
GABA	-0.2024	0.130	[-0.460, 0.055]	-1.562	0.122	0.183	8.1%
Glutamate	0.0622	0.131	[-0.198, 0.322]	0.476	0.635	0.714	2.5%
Histamine	-0.2043	0.098	[-0.399, -0.009]	-2.079	0.040	0.122	8.2%
Norepinephrine	0.4667	0.142	[0.184, 0.749]	3.282	0.001	0.009	18.6%
Opioid	0.3170	0.160	[-0.001, 0.635]	1.977	0.051	0.122	12.7%
Serotonin	0.4862	0.216	[0.057, 0.916]	2.249	0.027	0.122	19.4%

Supplementary Table 3. Multivariate linear regression prediction parameters of nine neurotransmitters.

Neurotransmitter	VIF
Acetylcholine	8.09
Cannabinoid	4.51
Dopamine	4.06
GABA	3.94
Glutamate	3.99
Histamine	2.68
Norepinephrine	5.00
Opioid	7.11
Serotonin	4.35

Supplementary Table 4. variance inflation factors (VIFs) for the nine neurotransmitters.

Neurotransmitter	weights	Contribution (%) Mean±SE	95% CI	*P*	P_{FDR}
Acetylcholine	-0.27	15.74±6.36	[-0.50, 0.06]	0.047	0.102
Cannabinoid	-0.12	6.66±5.41	[-0.30, 0.07]	0.328	0.422
Dopamine	-0.06	3.37±4.55	[-0.21, 0.10]	0.577	0.646
GABA	-0.19	10.91±4.40	[-0.34, 0.04]	0.068	0.102
Glutamate	0.05	2.95±6.48	[-0.16, 0.28]	0.646	0.646
Histamine	-0.20	11.27±4.84	[-0.35, 0.02]	0.035	0.102
Norepinephrine	0.36	20.65±5.49	[0.18, 0.55]	0.001	0.009
Opioid	0.25	14.30±6.55	[0.03, 0.48]	0.064	0.102
Serotonin	0.25	14.14±5.65	[0.05, 0.44]	0.026	0.102

Supplementary Table 5. Ridge regression prediction parameters of nine neurotransmitters..

Metric	Value
Median BF_{01}	0.83
$BF_{01} > 1$ (favoring null)	30%
$BF_{01} \geq 3$ (moderate or stronger evidence for null)	8%
$BF_{01} > 0.33$ (not providing strong evidence for a difference)	73%
$BF_{01} < 1/3$ (supporting a difference)	27%

Supplementary Table 6. Bayesian analysis statistics based on range.

	LEUVEN_2	NYU	OLIN	PITT	SDSU	TRINITY	UCLA_1	UM_1	UM_2	USM
**ASD (n)**	14	22	13	11	14	13	20	25	10	20
**TD (n)**	19	36	8	12	20	13	18	27	12	8
**TR (ms)**	1667	2000	1500	1500	2000	2000	3000	2000	2000	2000
**Field Strength**	3T	3T	3T	3T	3T	3T	3T	3T	3.0T	3T
**ASD_Age (yeasrs)**	13.7±1.2 (12.1–15.3)	14.4±1.5 (12.1–17.9)	15.1±2.1 (12.0–18.0)	14.2±1.7 (12.0–17.0)	14.7±1.8 (12.1–17.2)	14.8±1.5 (12.0–17.2)	14.8±1.6 (12.3–17.9)	14.3±1.6 (12.0–18.0)	14.5±1.4 (12.8–16.6)	16.3±1.1 (12.3–17.8)
**TD_Age (years)**	14.5±1.5 (12.2–16.9)	14.6±1.7 (12.1–17.7)	15.2±1.5 (13.0–17.0)	14.6±1.9 (12.2–17.4)	14.4±1.6 (12.1–16.9)	14.7±1.8 (12.0–17.5)	14.7±1.7 (12.4–17.8)	15.6±1.8 (12.2–18.0)	15.2±1.7 (13.3–17.9)	15.3±1.4 (12.5–17.1)
**Age *p***	0.124	0.642	0.903	0.617	0.628	0.875	0.842	0.012*	0.314	0.087
**ASD_IQ**	94.6±18.2	82.7±42.6	109.8±17.5	108.1±9.8	112.4±18.3	106.2±13.7	101.5±11.3	102.8±15.7	118.6±9.2	78.5±49.2
**TD_IQ**	108.5±12.4	78.5±22.3	105.6±13.8	108.5±9.2	108.6±11.2	107.8±15.2	100.8±9.6	108.3±13.5	112.4±10.5	82.6±52.7
**IQ *p***	0.037*	0.618	0.542	0.926	0.451	0.776	0.845	0.174	0.182	0.852
**ASD_FD**	0.19±0.08 (0.10–0.39)	0.14±0.05 (0.05–0.25)	0.32±0.23 (0.14–2.09)	0.21±0.07 (0.09–0.35)	0.18±0.26 (0.05–1.03)	0.19±0.23 (0.10–0.97)	0.21±0.24 (0.07–1.10)	0.41±0.58 (0.06–2.21)	0.17±0.12 (0.06–0.85)	0.31±0.28 (0.06–1.20)

	LEUVEN_2	NYU	OLIN	PITT	SDSU	TRINITY	UCLA_1	UM_1	UM_2	USM
**TD_FD**	0.16±0.04 (0.10–0.24)	0.11±0.07 (0.06–0.35)	0.32±0.25 (0.13–0.79)	0.21±0.05 (0.14–0.31)	0.12±0.06 (0.06–0.25)	0.18±0.21 (0.10–0.84)	0.16±0.15 (0.07–0.72)	0.12±0.11 (0.06–0.54)	0.19±0.24 (0.07–0.79)	0.21±0.08 (0.12–0.38)
**FD *p***	0.168	0.083	0.985	0.973	0.342	0.901	0.452	0.021*	0.812	0.341
**ASD_ Sex (M/F),**	10/4	16/6	11/2	9/2	14/0	13/0	18/2	23/2	9/1	20/0
**TD_ Sex (M/F),**	11/8	25/11	6/2	10/2	18/2	13/0	16/2	22/5	10/2	8/0
**Sex *p***	0.512	0.891	0.672	0.912	0.227	1.000	0.813	0.241	0.781	1.000
**ASD_ Handedness (R/L/M),**	12/2/0	22/0/0	11/2/0	11/0/0	13/1/0	13/0/0	20/0/0	24/1/0	10/0/0	20/0/0
**TD_ Handedness (R/L/M)**	15/4/0	36/0/0	7/1/0	12/0/0	18/2/0	13/0/0	18/0/0	25/2/0	12/0/0	8/0/0
**Handedness *p***	0.721	1.000	0.883	1.000	0.892	1.000	1.000	0.792	1.000	1.000

Supplementary Table 7. Site-specific demographic characteristics, head motion, and group matching statistics for adolescents with autism spectrum disorder (ASD) and typically developing (TD) participants Data are presented as mean ± standard deviation (range) for continuous variables and as frequencies for categorical variables. Group

differences were analyzed using independent-samples t-test or Mann–Whitney U test for continuous data, and chi-squared test for categorical data. TR, repetition time; FIQ, full-scale intelligence quotient; FD, framewise displacement; M, male; F, female; R, right; L, left; M, mixed. Asterisks indicate significant group differences ($p < 0.05$).

Supplementary method: Convergence condition of Sill

Convergence condition: Set the iteration step size to be less than the fitting process. If any vertex meets the following conditions, it is determined as a fitting failure: (1) The number of iterations exceeds 5000 times and still does not converge; (2) The fitting parameters (Sill or Range) exceed the reasonable range defined in advance (Sill < 0 or Sill > 1, or Range < 1 mm or Range > 200 mm)

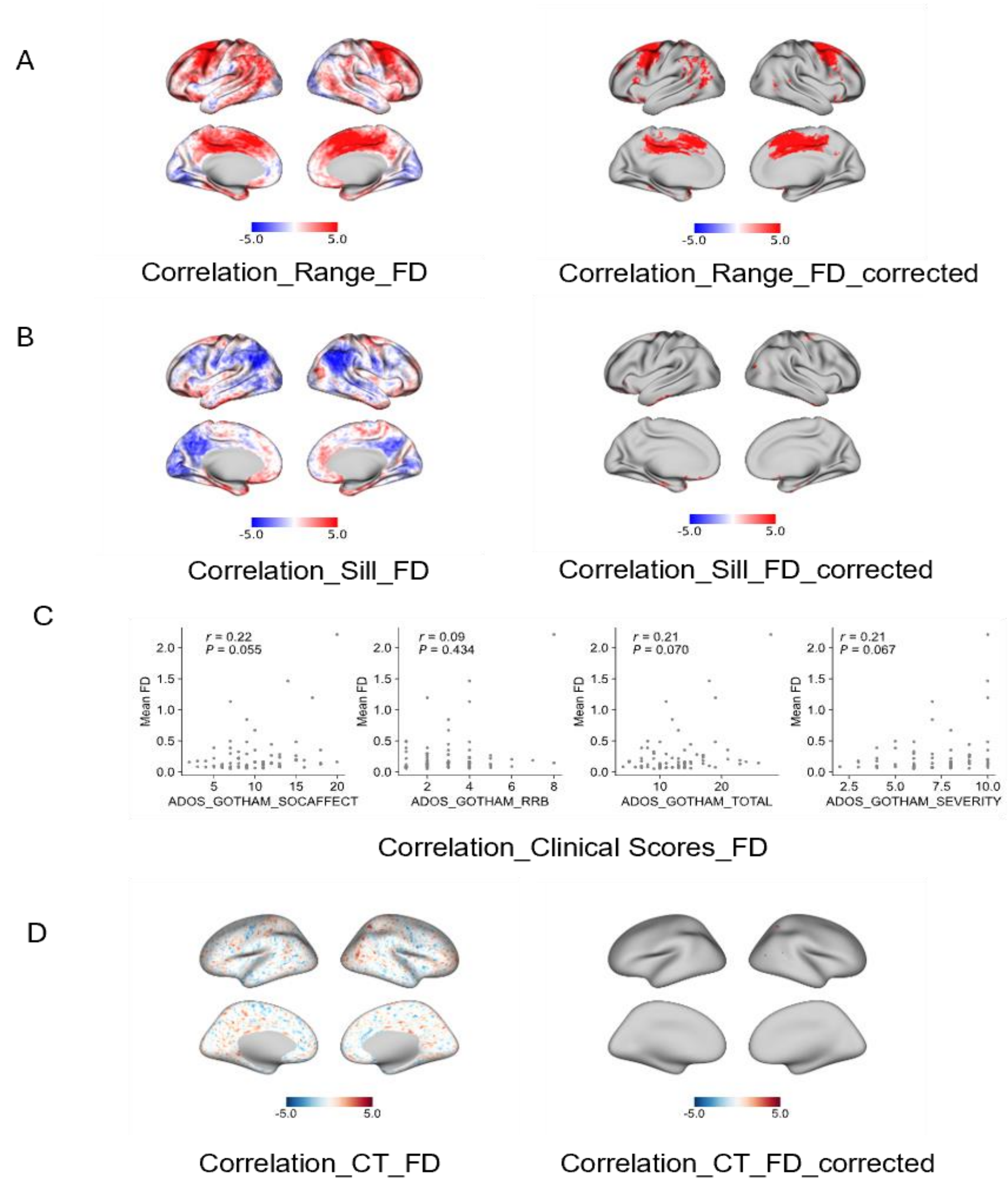
A
-5.0
5.0
Correlation_Range_FD
-5.0
5.0
Correlation_Range_FD_corrected
B
-5.0
5.0
Correlation_Sill_FD
-5.0
5.0
Correlation_Sill_FD_corrected
C
r = 0.22
P = 0.055
Mean FD
ADOS_GOTHAM_SOCAFFECT
r = 0.09
P = 0.434
Mean FD
ADOS_GOTHAM_RRB
r = 0.21
P = 0.070
Mean FD
ADOS_GOTHAM_TOTAL
r = 0.21
P = 0.067
Mean FD
ADOS_GOTHAM_SEVERITY
Correlation_Clinical Scores_FD
D
-5.0
5.0
Correlation_CT_FD
-5.0
5.0
Correlation_CT_FD_corrected

Supplementary Figure S1. Correlations of mean framewise displacement (FD) with spatial-functional metrics, cortical thickness, and clinical symptom scores.

A) Correlations between FD and Range. Vertex-wise maps before (left) and after (right) FDR correction show scattered associations, but no corrected clusters overlapped with the main ASD–TD difference regions.

B) Correlations between FD and Sill. Vertex-wise maps before (left) and after (right) FDR correction similarly show no significant clusters overlapping with our main effects.

C) Correlations between FD and clinical symptom scores. Scatter plots of mean FD against ADOS-Gotham social-affective, restricted/repetitive behavior, total, and severity scores, all of which were non-significant.

D) Correlations between FD and cortical thickness. Vertex-wise maps before (left) and after (right) FDR correction show no significant clusters overlapping with the main ASD–TD difference maps.

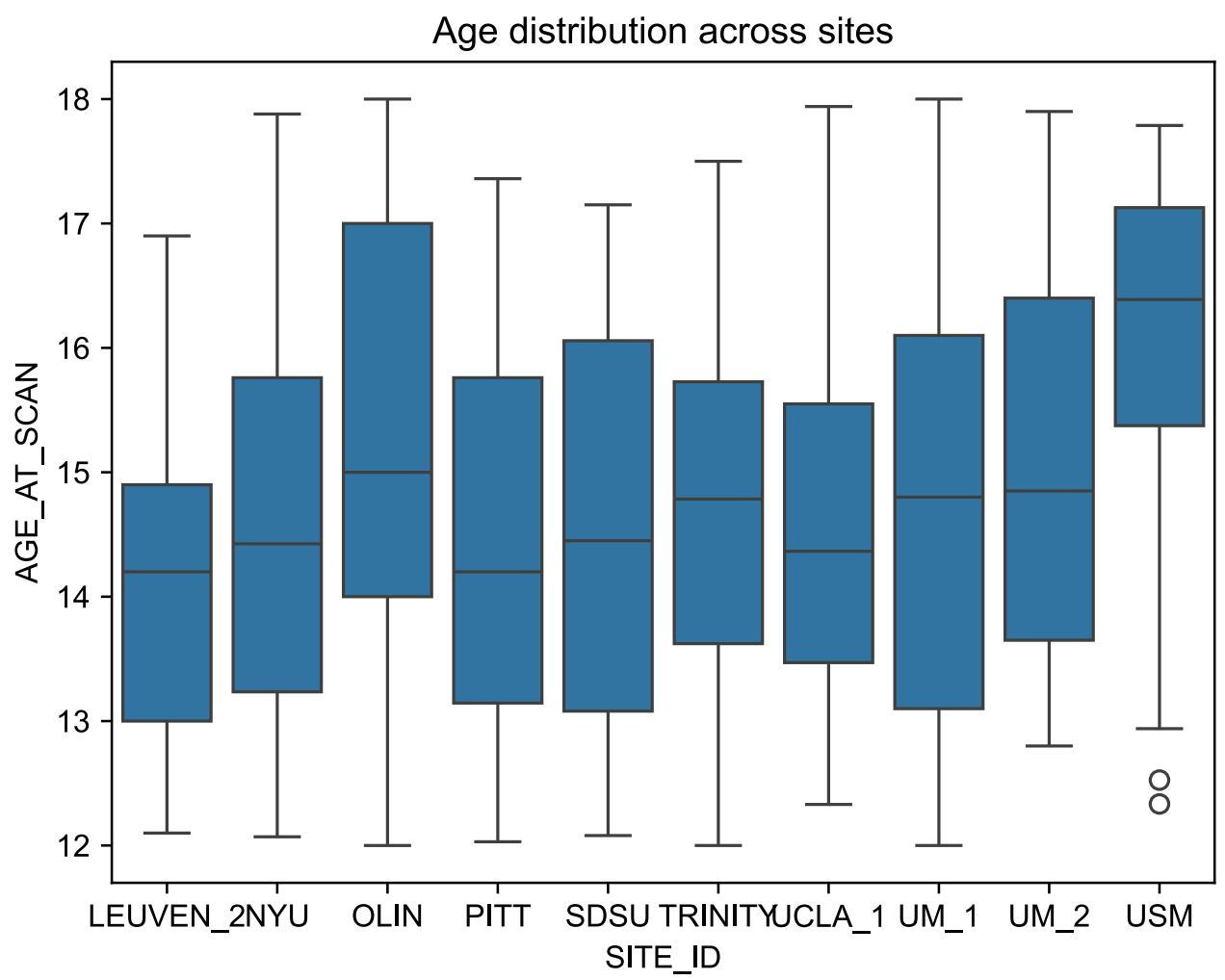

Supplementary Figure S2. Age distribution across data collection sites. Boxplots showing the distribution of participant age (in years at scan) for each site included in the analysis. The horizontal line within each box indicates the median age, the box spans the interquartile range (25th–75th percentiles), and the whiskers extend to the minimum and maximum values within 1.5 times the interquartile range. An outlier is marked for the USM site.

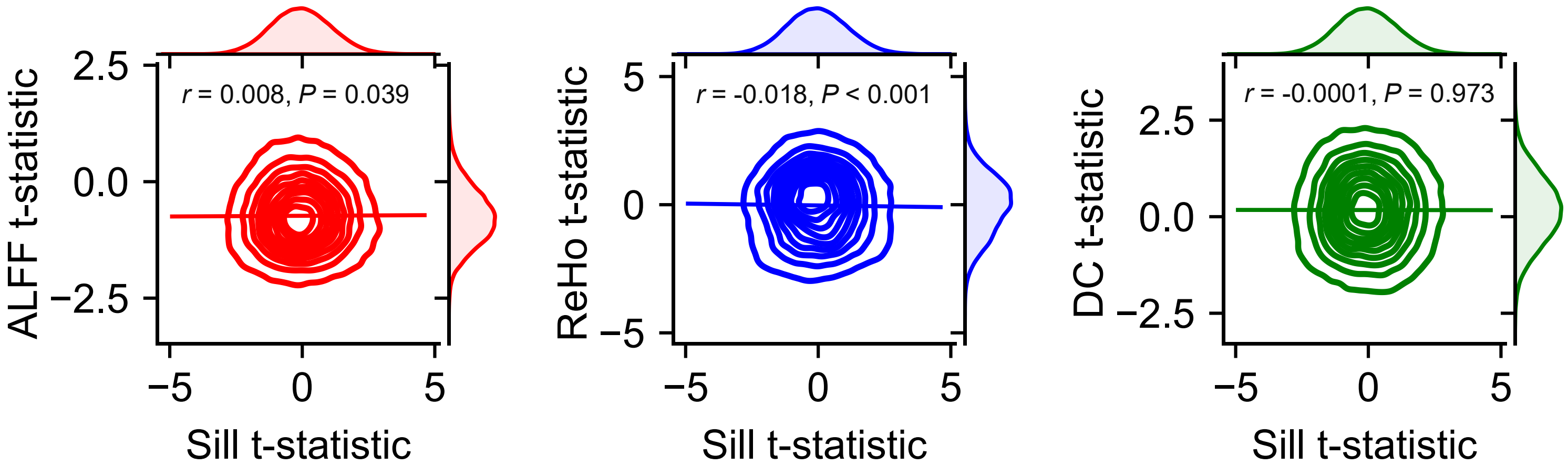

Supplementary Figure S3. Spatial correlations between variogram-derived metrics and conventional rs-fMRI measures. Vertex-wise t-statistic maps for ASD vs. TD differences were computed for Sill, Range, ReHo, ALFF, and degree centrality (DC). The spatial distributions of Sill t -statistic alterations exhibited weak/non-significant correlations with the patterns observed for these conventional measures.

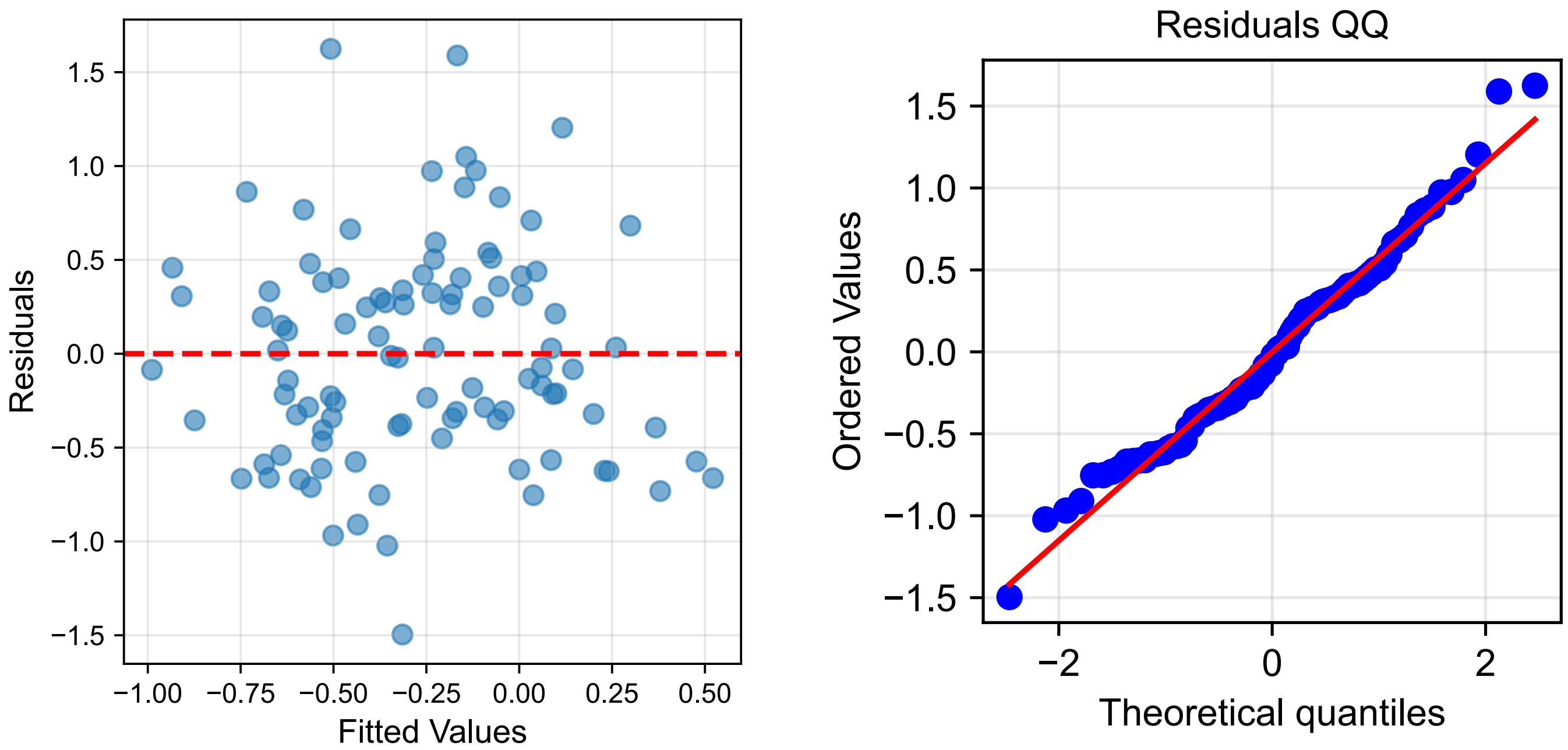

Supplementary Figure S4. Residual plots for the multiple linear regression model. The residuals show approximately random distribution around zero wit h no systematic heteroscedasticity.

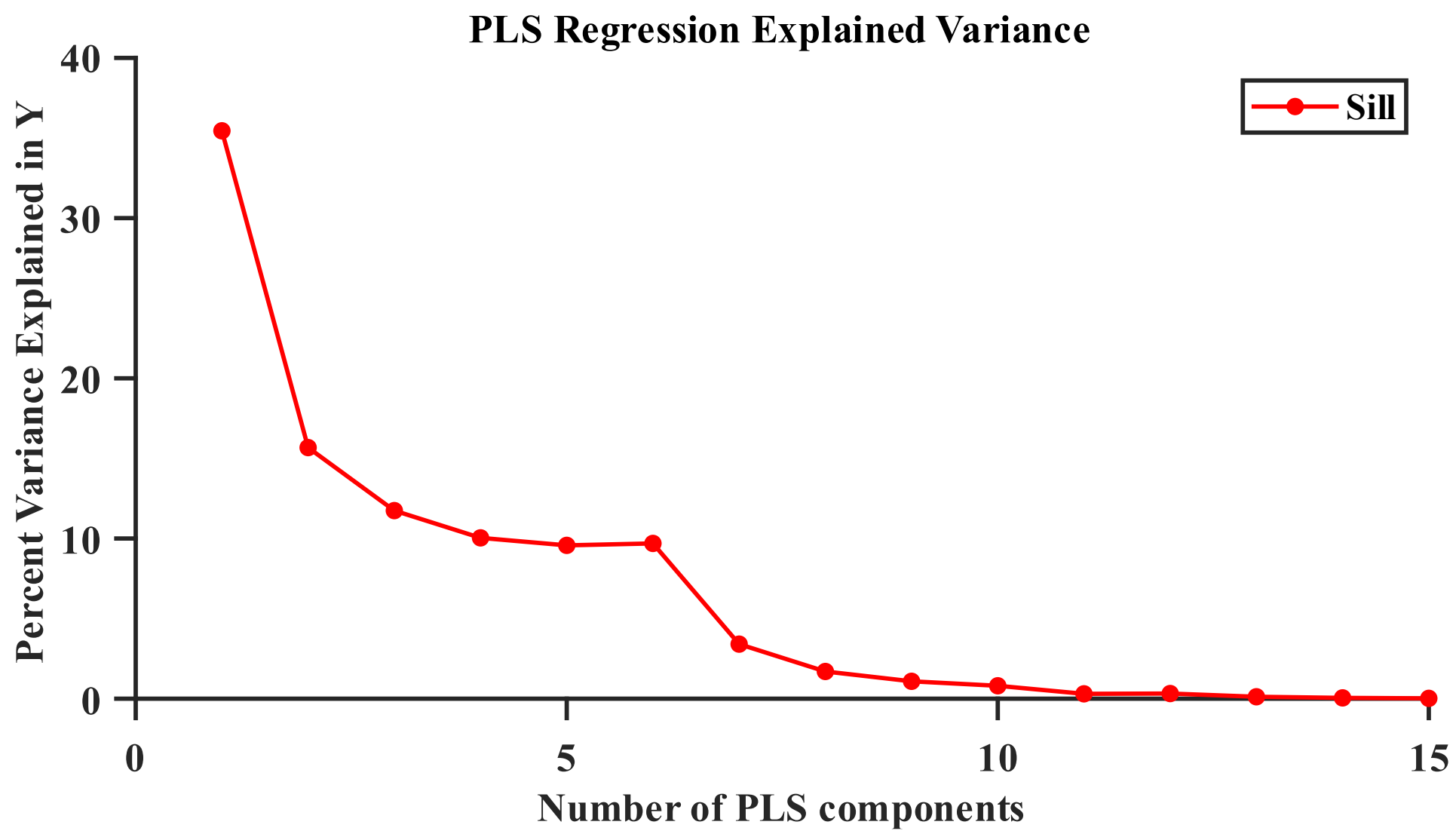

Supplementary Figure S5. The scree plot of variance explained across all PLS components confirms that PLS1 represents the dominant latent component, with subsequent components explaining substantially less variance.

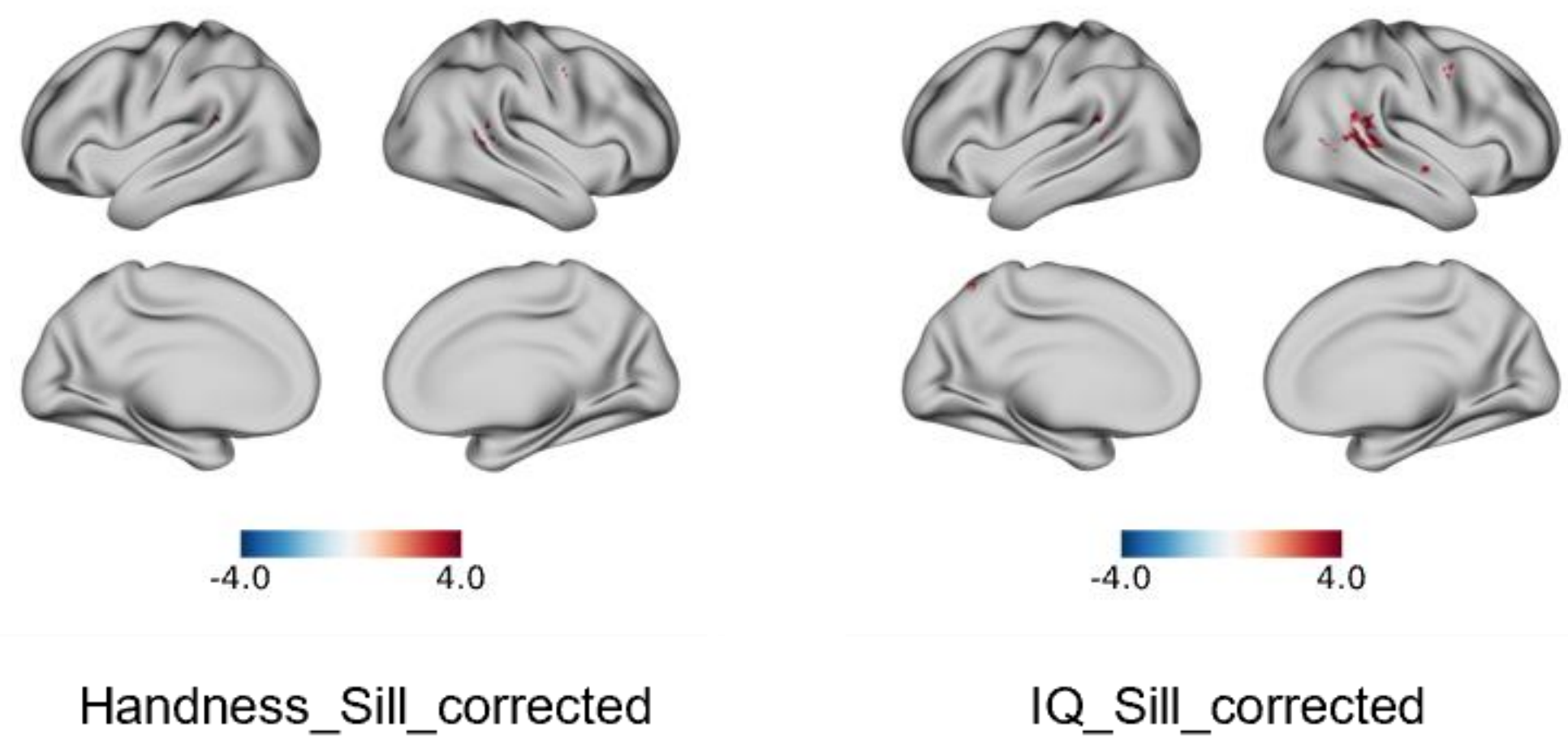

Supplementary Figure S6. Control analyses of handedness and IQ in relation to Sill. Vertex-wise correlation analyses between Sill values and handedness (left) and full-scale IQ (right), both after correction for multiple comparisons. After correction for multiple comparisons, no clusters over lapped with the primary ASD–TD Sill difference regions reported in the main analysis.

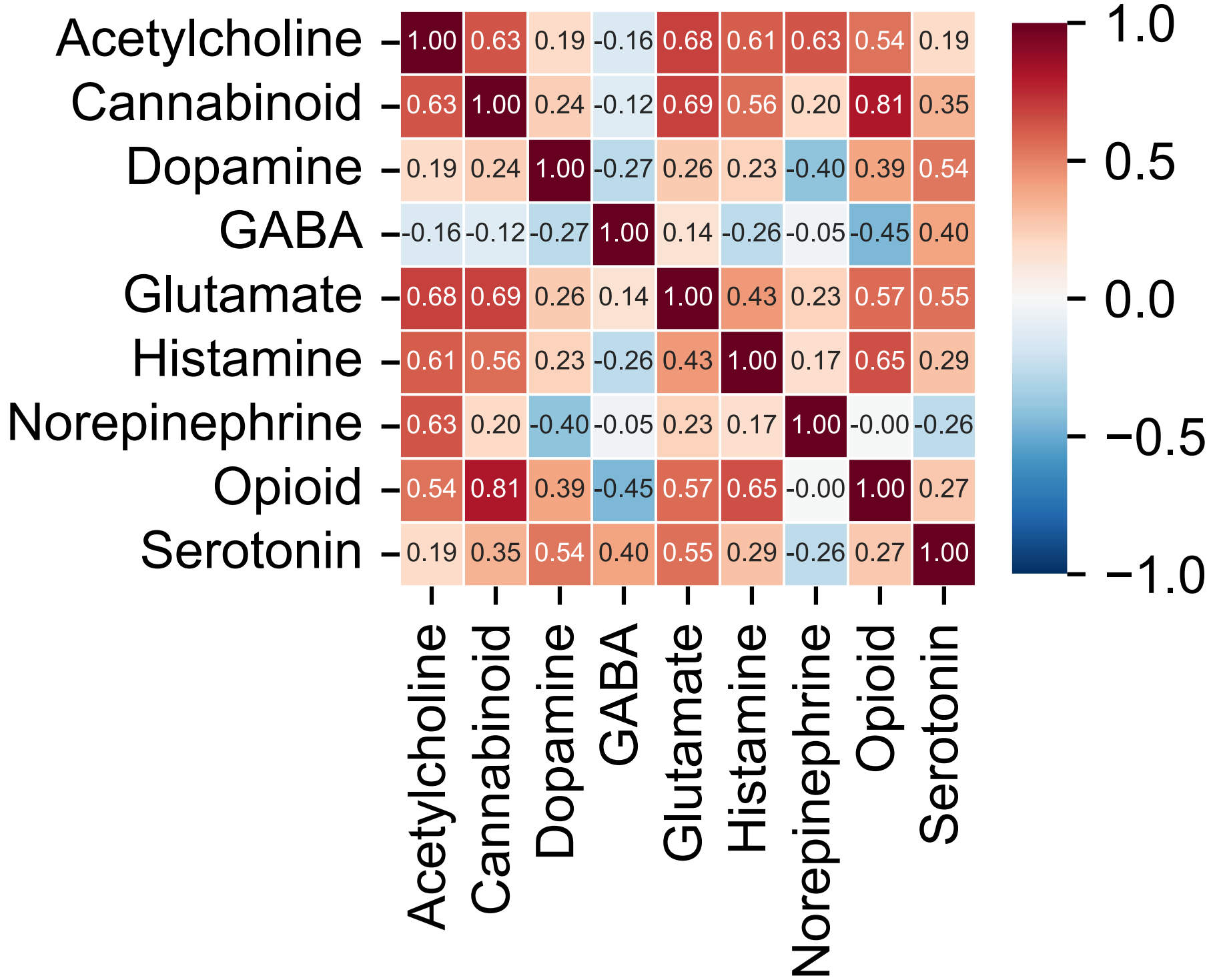

Supplementary Figure S7. Pairwise correlation matrices for the nine neurotransmitters.

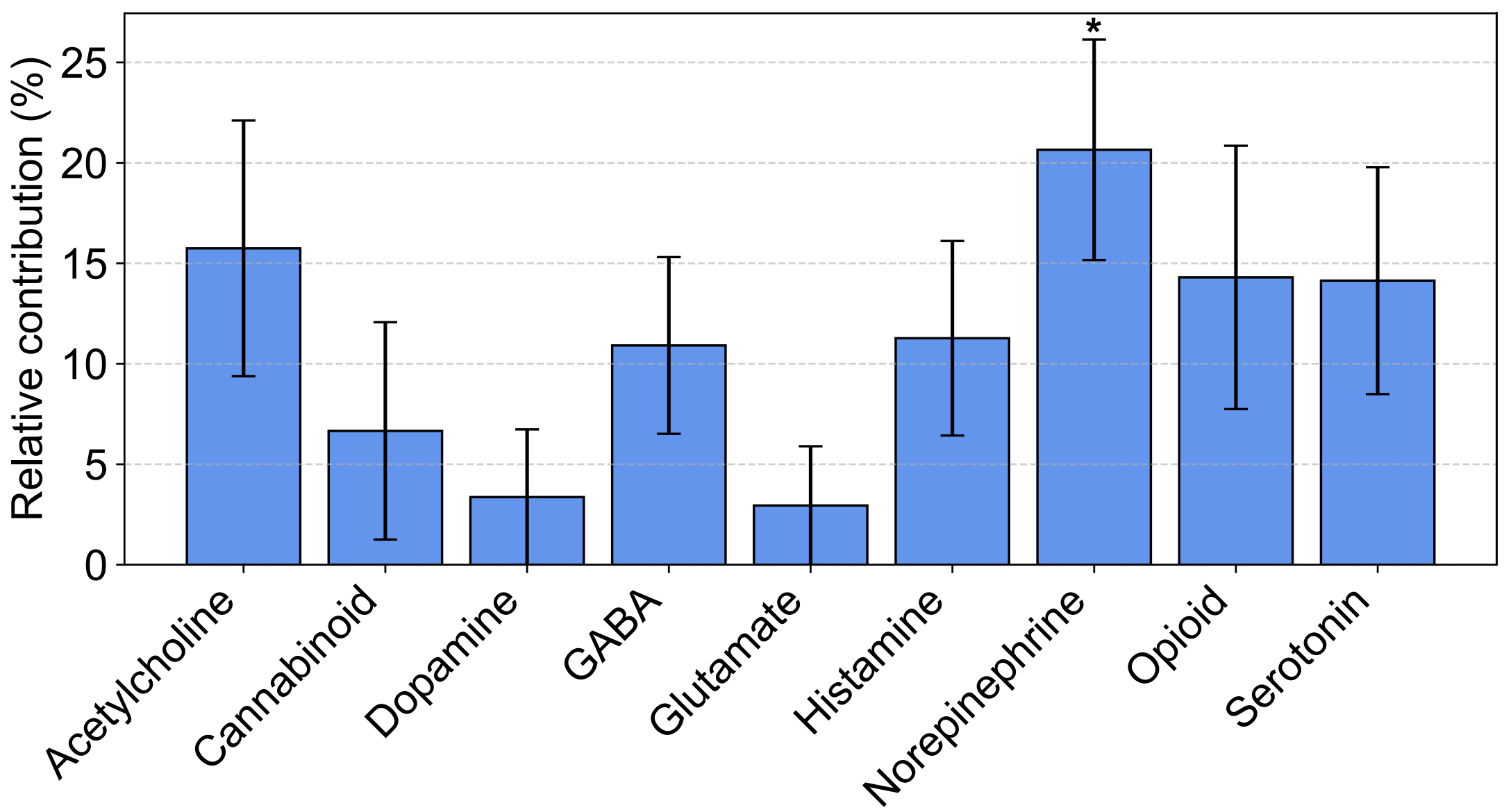

Supplementary Figure S8. Ridge Regression Analysis for nine neurotransmitters.

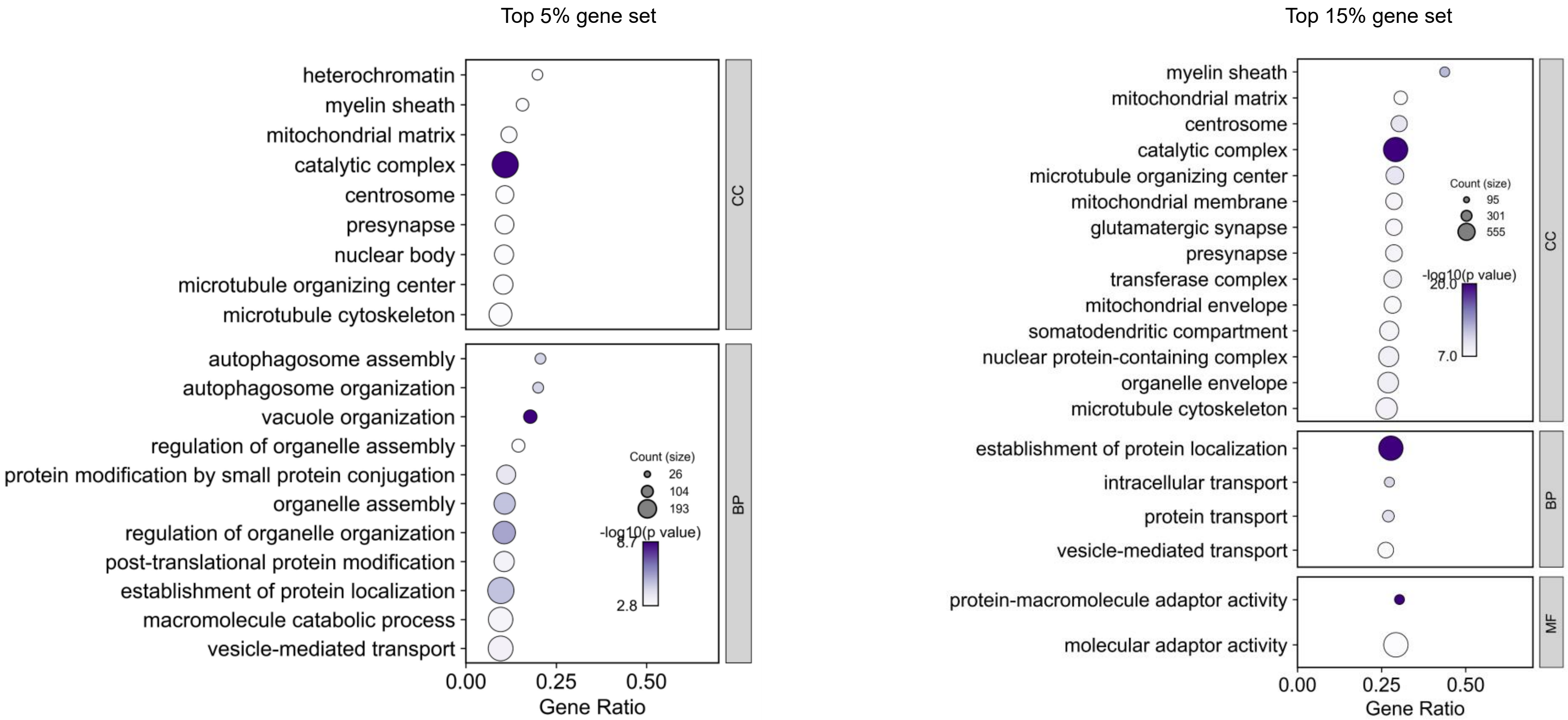

Supplementary Figure S9. Robustness of enrichment results across alternative gene-weight thresholds. Gene Ontology enrichment analysis was performed on PLS+ and PLS− gene sets defined by the top/bottom 5% and 15% of PLS1 weights. Bubble size represents gene count; color indicates enrichment significance ($-\log_{10}$(FDR)). The myelin sheath, mitochondrial, and synaptic categories showed consistent enrichment across all three thresholds, supporting the robustness of the 10% cutoff selection.

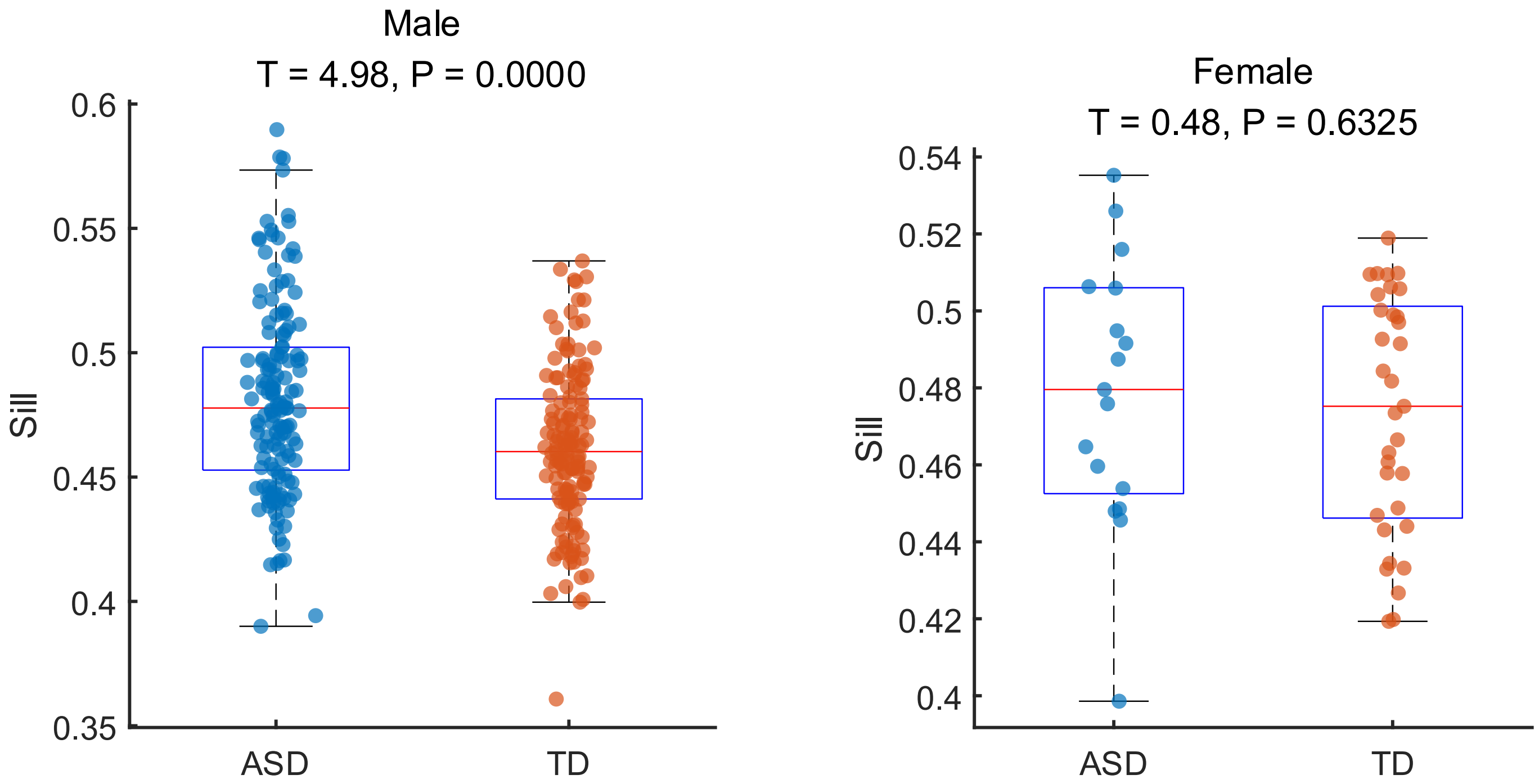

Supplementary Figure S10. Sex-stratified comparison of Sill differences between ASD and TD groups. Boxplots illustrate the distribution of Sill values across four subgroups: male TD (132), male ASD (132), female TD (44), and female ASD (31). In the male subgroup, ASD participants showed significantly elevated Sill values compared to TD participants ($p < 0.05$, corrected). In the female subgroup, the same directional trend (higher Sill in ASD) was observed but did not reach statistical significance, likely due to the smaller sample size of female participants in the ABIDE-I dataset. Whiskers represent the full range of values, and boxes represent the interquartile range with median indicated by the center line.

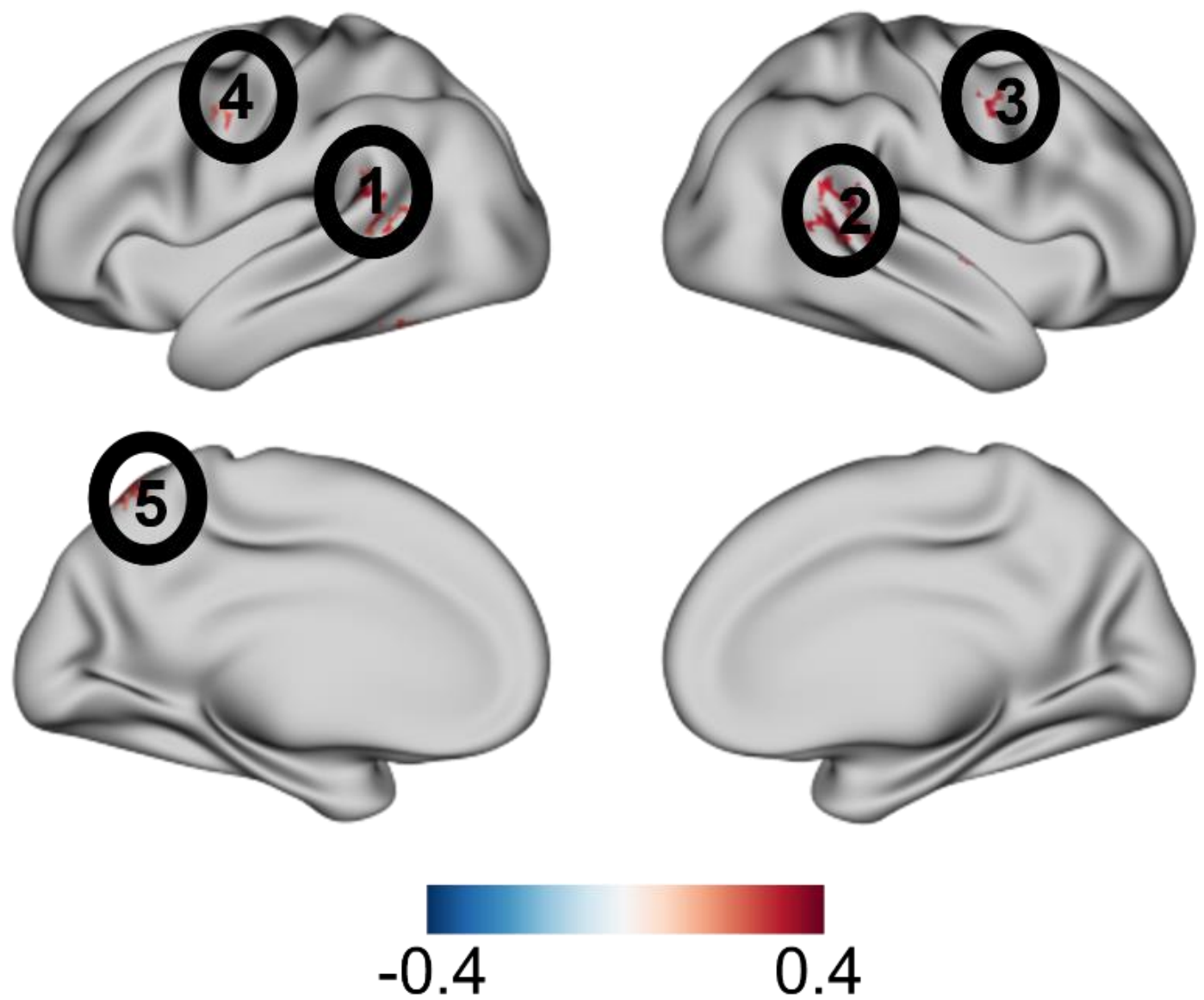

Region	ASD (Mean±SD)	TD (Mean±SD)	t	*p*	Cohen's d
1	0.507±0.038	0.489±0.034	4.63	5.12×10−6	0.50
2	0.466±0.043	0.453±0.032	3.15	1.72×10−3	0.34
3	0.498±0.029	0.483±0.041	3.88	1.22×10−4	0.42
4	0.492±0.044	0.476±0.033	3.73	2.10×10−4	0.42
5	0.478±0.039	0.459±0.031	4.98	8.35×10−7	0.54

Supplementary Figure S11. ASD-TD Sill differences map with global signal regression. The ASD–TD comparison was performed using vertex-wise two-sample t-tests.
